# Emergence of the *Dickeya* genus involved duplication of the OmpF porin and the adaptation of the EnvZ-OmpR signaling network

**DOI:** 10.1128/spectrum.00833-23

**Published:** 2023-08-29

**Authors:** Clémence Cochard, Marine Caby, Peggy Gruau, Edwige Madec, Michael Marceau, Iulia Macavei, Jérôme Lemoine, Chrystelle Le Danvic, Franck Bouchart, Brigitte Delrue, Sébastien Bontemps-Gallo, Jean-Marie Lacroix

**Affiliations:** 1 Univ. Lille, CNRS, UMR 8576 - UGSF - Unité de Glycobiologie Structurale et Fonctionnelle, Lille, France; 2 Univ. Lille, CNRS, Inserm, CHU Lille, Institut Pasteur de Lille, U1019 - UMR 9017 - CIIL - Center for Infection and Immunity of Lille, Lille, France; 3 Univ. Lyon, CNRS, Université Claude Bernard Lyon 1, Institut des Sciences Analytiques, UMR 5280, Villeurbanne, France; 4 R&D Department, ALLICE, Paris, France; 5 Université Polytechnique Hauts-de-France, EA 2443 - LMCPA - Laboratoire des Matériaux Céramiques et Procédés Associés, Valenciennes, France; Institute of Parasitology, Biology Centre, ASCR, Ceske Budejovice, Czech Republic

**Keywords:** *Dickeya dadantii*, EnvZ-OmpR, two-component system, porin, plant pathogen, phenolic acids

## Abstract

Genome evolution, and more specifically gene duplication, is a key process shaping host–microorganism interaction. The conserved paralogs usually provide an advantage to the bacterium to thrive. If not, these genes become pseudogenes and disappear. Here, we show that during the emergence of the genus *Dickeya*, the gene encoding the porin OmpF was duplicated. Our results show that the *ompF2* expression is deleterious to the virulence of *Dickeya dadantii*, the agent causing soft rot disease. Interestingly, *ompF*2 is regulated while *ompF* is constitutive but activated by the EnvZ-OmpR two-component system. *In vitro*, acidic pH triggers the system. The pH measured in four eudicotyledons increased from an initial pH of 5.5 to 7 within 8 h post-infection. Then, the pH decreased to 5.5 at 10 h post-infection and until full maceration of the plant tissue. Yet, the production of phenolic acids by the plant’s defenses prevents the activation of the EnvZ-OmpR system to avoid the *ompF2* expression even though environmental conditions should trigger this system. We highlight that gene duplication in a pathogen is not automatically an advantage for the infectious process and that, there was a need for our model organism to adapt its genetic regulatory networks to conserve these duplicated genes.

**IMPORTANCE:**

*Dickeya* species cause various diseases in a wide range of crops and ornamental plants. Understanding the molecular program that allows the bacterium to colonize the plant is key to developing new pest control methods. Unlike other enterobacterial pathogens, *Dickeya dadantii*, the causal agent of soft rot disease, does not require the EnvZ-OmpR system for virulence. Here, we showed that during the emergence of the genus *Dickeya*, the gene encoding the porin OmpF was duplicated and that the expression of *ompF2* was deleterious for virulence. We revealed that while the EnvZ-OmpR system was activated *in vitro* by acidic pH and even though the pH was acidic when the plant is colonized, this system was repressed by phenolic acid (generated by the plant’s defenses). These results provide a unique— biologically relevant—perspective on the consequence of gene duplication and the adaptive nature of regulatory networks to retain the duplicated gene.

## INTRODUCTION

*Dickeya dadantii* is a model phytopathogenic bacterium that caused soft rot disease and massive economic losses in agriculture (1). Attracted by the jasmonic acid secreted by the plant upon wounding, *D. dadantii* invades the mesophyll tissues (apoplast). In this nutrient-limited and highly stressful environment, the bacterium must quickly protect itself from this environment to initiate host colonization. If the bacterium overcomes the plant’s defenses, the symptomatic phase begins, marked by the appearance and extension of maceration due to the massive secretion of plant cell wall degrading enzymes (2). During the infection, *D. dadantii* must deal with several environmental changes (stresses) including plant defenses, pH, and osmolarity.

Two-component systems (TCSs) also called phosphorelays are the main system for sensing and adaptation to changing environments. Under the specific stimulus, a membrane sensor is activated, autophosphorylates onto a conserved histidine residue, and then transfers its phosphate group onto a conserved aspartate residue of its cognate transcriptional regulator. The ratio of the phosphorylated regulator to the overall regulator enables the fine modulation of hindered sets to respond to the sensed stimulus (3). Forty years of research on the archetypal EnvZ-OmpR system has enabled us to characterize the transfer of phosphate from the sensor to its regulator, the regulation of porins (e.g., OmpF) by osmolarity via this system (4 – 6), and its involvement in the regulation of biofilms, motility, and virulence factors (7, 8). Required for virulence in human pathogens such as *Salmonella enterica* (9) or *Yersinia pestis* (10, 11), the role of EnvZ-OmpR in plant pathogens remains unclear. In the fire blight pathogen, *Erwinia amylovora*, this TCS was not required for virulence (8) while in the soft rot pathogen, *D. dadantii*, an *ompR* mutant strain survival is better than the wild-type strain in the pea aphid (12) but is fully virulent in the plant (13). This discrepancy in the requirement of the EnvZ-OmpR system for effective virulence prompts us to decipher the role of this system in the pathogenesis of *D. dadantii*.

In this study, we demonstrate that this system is activated *in vitro* by acidic pH but not osmolarity. *In planta*, while the pH is acidic, the EnvZ-OmpR system is silenced by the production of phenolic compounds, a common response in plants exposed to pathogens. Comparative genome analyses of different plant pathogens revealed that the *ompF* gene has been duplicated in the *Dickeya* genus and that both *ompF* and *ompF2* are expressed at acidic pH through activation of the EnvZ-OmpR system. Interestingly, the *ompF2* expression is deleterious for virulence and required inactivation of the EnvZ-OmpR system for successful plant colonization. These results not only demonstrate that the shutdown of EnvZ-OmpR activation represents a critical step for the plant invasion but also provide important insight into the plant–bacteria interactions that illustrate the broader biological significance of specific integration of the two-component system into the genetic network of a bacterial pathogen.

## RESULTS

### The EnvZ-OmpR system is dispensable for in planta virulence of *D. dadantii*

**Fig 1:**
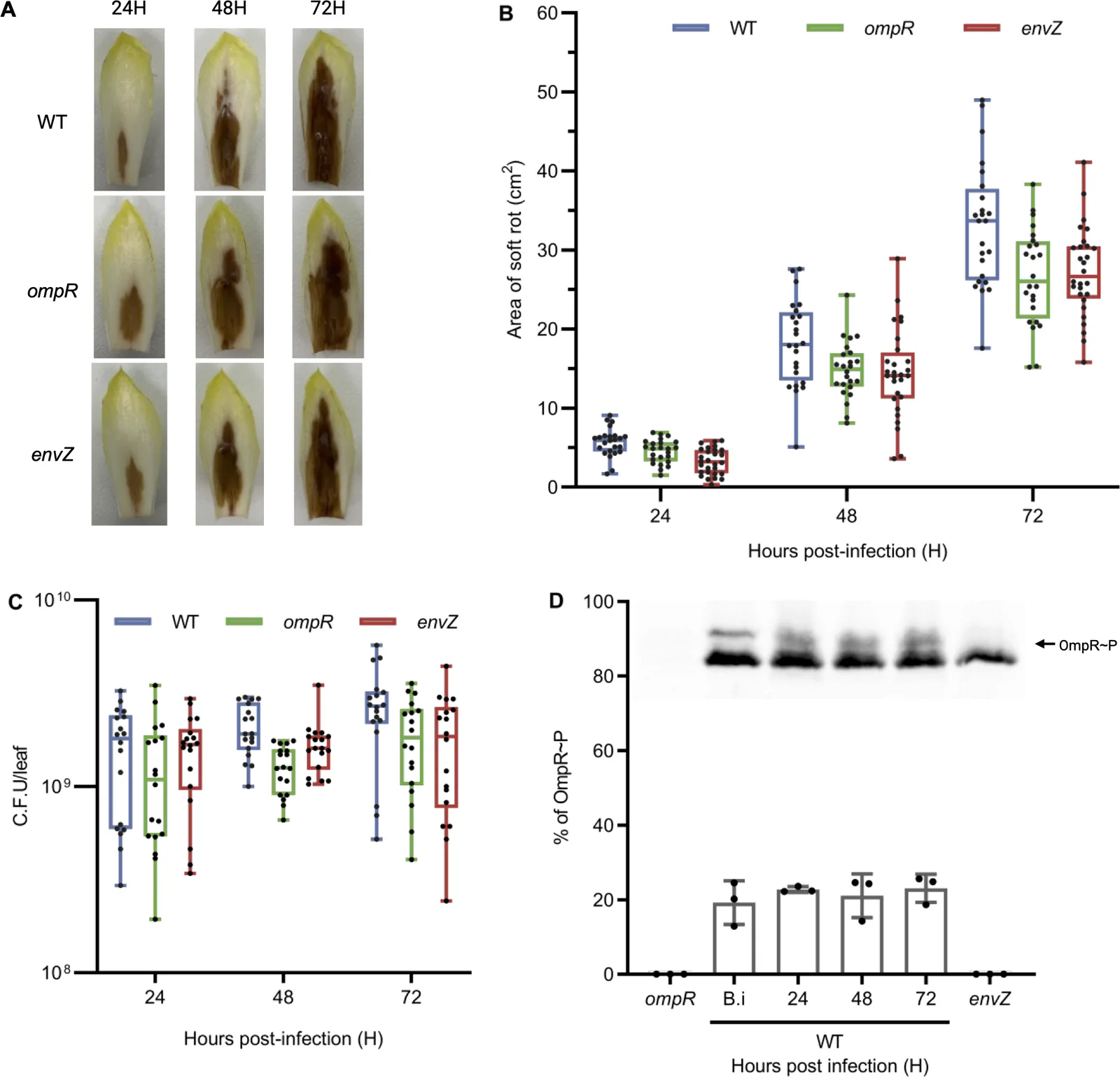
The EnvZ-OmpR system is dispensable for virulence. (**A**) Representative time course of symptom development for the wild-type, *ompR*, *envZ* strains inoculated into chicory leaves. Virulence was monitored for 3 d. Extension of macerated area (**B**) and bacterial load (colony forming unit) (**C**) for the wild-type, *ompR*, and *envZ* strains inoculated into chicory leaves (*n* = 6). Experiments were repeated three times. (**D**) Representative immunoblot of the separation of OmpR and OmpR~*P* by SDS-PAGE Phos-Tag gel after extraction of bacteria during the infectious cycle and quantification of OmpR and OmpR~P. Cell lysate of wild type before infection and after 24, 48, and 72 h post-infection, *envZ* and *ompR* mutants from exponential growth phase culture of *D. dadantii* were loaded into SDS-PAGE Phos-Tag gel. Both forms of OmpR were revealed by Western blot (*n* = 3 biological replicates).

Based on previous observations showing that the *ompR* mutant survives better in the pea aphid than the wild-type strain (12), we hypothesized that the *envZ-ompR* mutant survives better *in planta*. To test this hypothesis, chicory leaves were infected with the wild-type strain, the *ompR,* and the *envZ* single mutant strains. As observed previously, no difference in the severity of the disease symptoms (i.e., the kinetics of maceration development and extent) could be observed (Fig. 1A and B). Daily bacterial enumeration up to 3 d post-infection did not reveal any increase in survival ability for the mutants (Fig. 1C). Our data indicate that EnvZ-OmpR does not contribute to the virulence process.

As observed in other bacteria (14 – 16), the EnvZ-OmpR signaling system could be related to bacterial fitness during *D. dadantii* infection. If so, the system activity level will adjust during the infection. In other words, the ratio of ompR phosphorylated form (OmpR~*P*)/OmpR total will be affected. We monitored the level of OmpR phosphorylation *in planta* over 3 d post-infection using a polyacrylamide Phos-Tag approach previously developed by our laboratory. Due to reversible binding with the Phos-Tag, the migration of the OmpR phosphorylated form relative to phosphorylated OmpR protein was delayed, allowing the separation and quantification of the phosphorylated fraction (upper band) versus the nonphosphorylated fraction (lower band). A fifth of OmpR was phosphorylated regardless of the condition (before infection, 24, 48, or 72 h) (Fig. 1D) indicating that the EnvZ-OmpR system activity remains constant during the *D. dadantii* infection cycle. Taken together, our data demonstrate that the EnvZ-OmpR system is not required for full virulence *in planta*.

### The pH transiently increases in planta at the early stage of the infection and can trigger the EnvZ-OmpR system

The EnvZ-OmpR two-component system activity is reported to be triggered by osmolarity and repressed by pH in other Enterobacteria (6). The current infection model of *D. dadantii* proposes that pH increases from 5 to more than 8 throughout the infection as soon as the bacteria penetrate the apoplast (2), while osmolarity increases due to the desiccation of the tissues (17). Therefore, these facts are inconsistent with our observation on the OmpR~*P*/OmpR ratio (Fig. 1D). This discrepancy leads us to hypothesize that (i) pH and osmolarity variation over the course of infection are not in accordance with the proposed model and (ii) concomitant pH and osmolarity fluctuations balance the kinase/phosphatase activities ratio of the EnvZ-OmpR system, which would explain our findings on OmpR phosphorylation rate in the plant (Fig. 1D).

**Fig 2:**
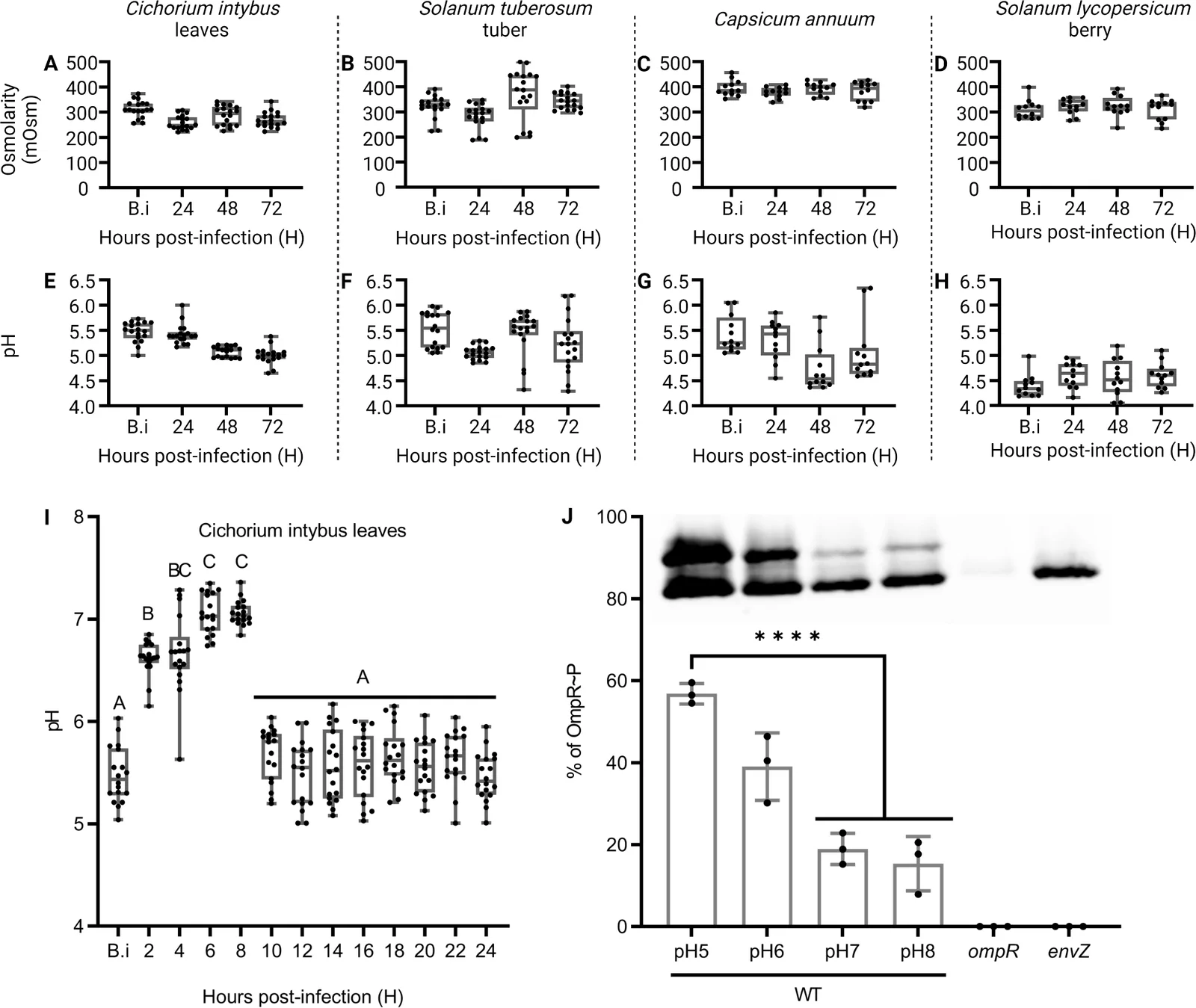
The pH transiently increases at the early stage of the infection and triggered the EnvZ-OmpR system. Tissue osmolarity and pH of chicory leaves (A, E, I), potatoes tuber (B and F), green pepper (C and G), and tomatoes (D and H) were measured with a nanoprobe pH sensor for pH and by vapor pressure osmometry for osmolarity (see Methods). Experiments were repeated three times. Groups with the same letter are not detectably different (*P* < 0.001, one-way ANOVA). (J) Representative immunoblot of the separation of OmpR and OmpR~*P* by SDS-PAGE Phos-Tag gel after extraction bacteria from exponential growth phase culture at different pH and quantification of OmpR and OmpR~P. Both forms of OmpR were revealed by Western blot (*n* = 3 biological replicates).

To specifically address the first question, osmolarity and pH were measured in four model organisms of agricultural interest: chicory leaves (*Cichorium intybus* var. *foliosum*), potato tuber (*Solanum tuberosum*), green pepper (*Capsicum annuum*), and tomato (*Solanum lycopersicum*). Tissue osmolarity was measured directly by freezing point osmometry. Surprisingly, regardless of the infection time and the species tested, the osmolarity remains constant (Fig. 2A through D). Measurement of pH was carried out using a microprobe pH meter every day for 3 d post-inoculation with the wild-type strain. Before infection, an acidic pH between 4.5 and 5.5 was observed (Fig. 2E through H). pH monitoring was extended up to 5 d post-inoculation, where leaves were macerated, and pH remained the same (data not shown). Therefore, it seems that pH remains constant during maceration. However, Nachin and Barras described an increase in pH within the first 24 h of infection using the pH indicator phenolsulfonphthalein (18). So, we decided to also monitor the pH at this asymptomatic stage of infection. Between 2 h and 8 h post-infection, the pH increased from 5.5 to 7, and then abruptly decreased to 5.5 at 10 h post-infection (Fig. 2I). These data refine the current model on stress encountered by *D. dadantii* during plant infection. Our findings also refute our hypothesis of a concomitant pH and osmolarity fluctuations balance of the OmpR~*P*/OmpR ratio.

To escape the complexity of the plant, we investigated the ability of pH to modulate *in vitro* the activation level of the EnvZ-OmpR. We analyzed, by polyacrylamide Phos-Tag gel, the effect of pH on EnvZ-OmpR activation level (Fig. 2J). At pH 8, only 11% ± 4 of OmpR was phosphorylated. A gradual increase in OmpR~*P* was measured when pH decreased. More than half (51% ± 5) of the OmpR were phosphorylated at pH 5 (Fig. 2J). We also confirmed that *in vitro*, osmolarity has no significant effect on the OmpR~*P*/OmpR ratio (Fig. S1). Taken together, our results outline a model where pH fluctuates in the early stages of infection and can trigger *in vitro* the EnvZ-OmpR system.

### Expression of OmpF requires EnvZ-dependent phosphorylation of OmpR

**Fig 3:**
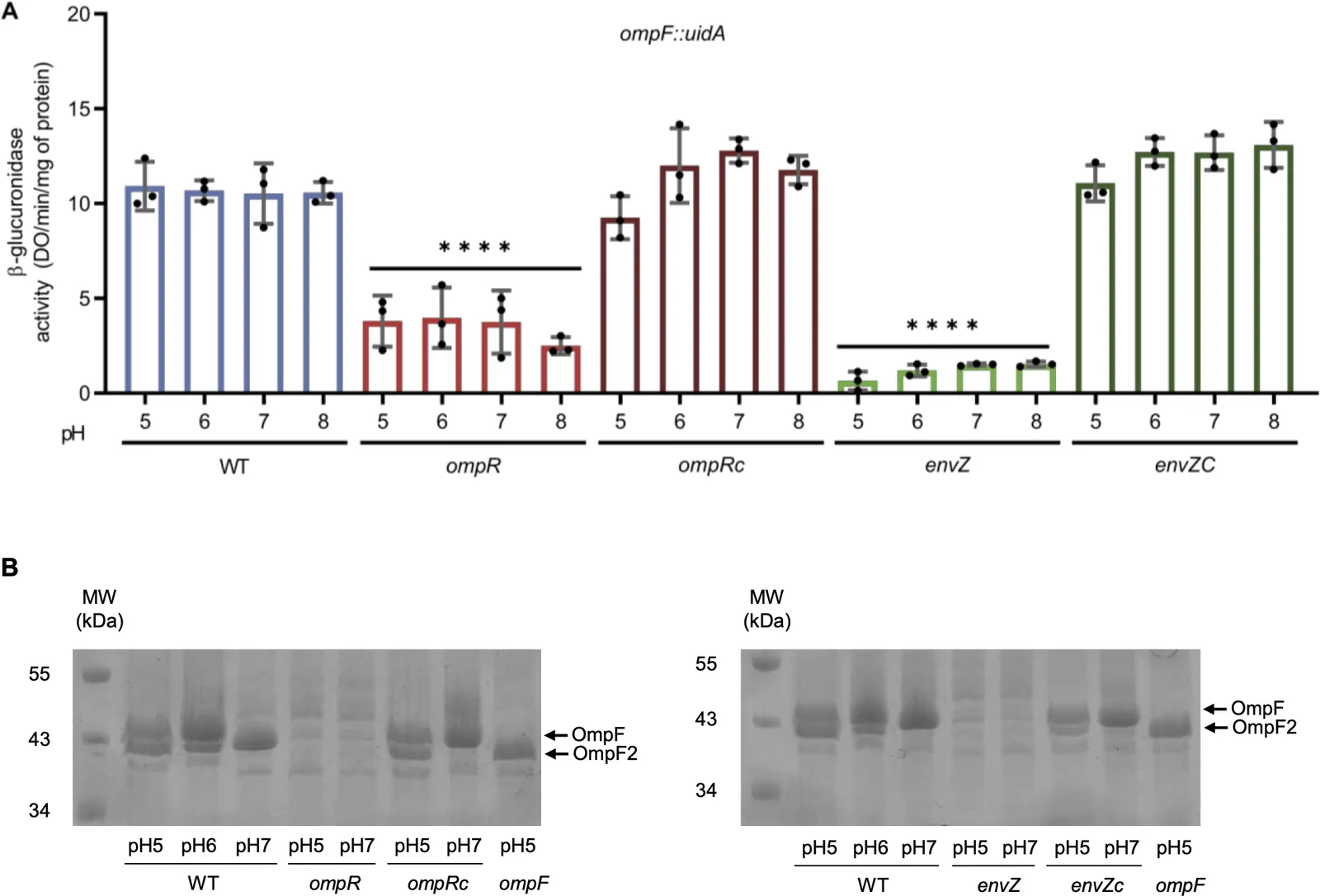
Expression of *ompF* requires EnvZ-dependent phosphorylation of OmpR. (**A**) Expression of *ompF::uidA* gene fusions at different pH in different genetic backgrounds. Bacteria were grown to the mid-log phase and lysed by sonication. The β-glucuronidase activity was measured with PNPU as a substrate. Specific activity was expressed as the change in OD_405_ per minute and per milligram of protein. The results are the average of three independent experiments. (**B**) Representative SDS-PAGE analysis of the outer membrane proteins showing the expression of the porin OmpF. A gel system supplied with 4 M urea was used and stained by Coomassie staining.

Yet, the acidic environment during infection should promote the activation of EnvZ-OmpR. This TCS is well known to regulate the balance between different porins to maintain an optimal amount in the envelope. *D. dadantii*’s genome harbored no *ompC* gene (19). The EnvZ-OmpR phosphorelay controls the expression of the porin *ompF* (20). Thus, assuming that porin is an essential constituent of the outer membrane of Enterobacteria and since OmpF seems to be the only porin in *D. dadantii*, *ompF* should be constitutively expressed during infection irrespectively of the pH value. To test this hypothesis, an *ompF::uidA* bioreporter was constructed, inserted by conjugation in strains of interest, and assessed at various pH. In the wild-type background, pH has no significant effect on *ompF* expression (Fig. 3A). In the *ompR* background, a basal expression of *ompF* was detected (30% of expression in the wild-type strain), indicating that OmpR is required for *ompF* gene expression (Fig. 3A). A similar basal expression was observed in the *envZ* strain (Fig. 3A) suggesting that in addition, a minimal amount of phosphorylated OmpR was required to allow expression of the *ompF* gene since the *envZ* strain harbors nondetectable phosphorylation of OmpR protein (Fig. 2J). The complementation of both *envZ* and *ompR* strains led to the restoration of the *ompF* gene expression (Fig. 3A).

To confirm these results at the protein level, the outer membrane proteins were isolated at various pH. No increase in the OmpF porin was detected when pH increased from 5 to 7 in the wild-type strain (Fig. 3B). In an *ompR* or *envZ* background, only a faint band was detected at all pH confirming the slight expression in these contexts of the *ompF::uidA* bioreporter. Complementation of both *envZ* and *ompR* strains restores the wild-type profile (Fig. 3B). These data confirmed that *ompF* expression is constitutive but requires activation by OmpR.

### The *Dickeya* genomes harbor three successive porin-encoded genes

**Fig 4:**
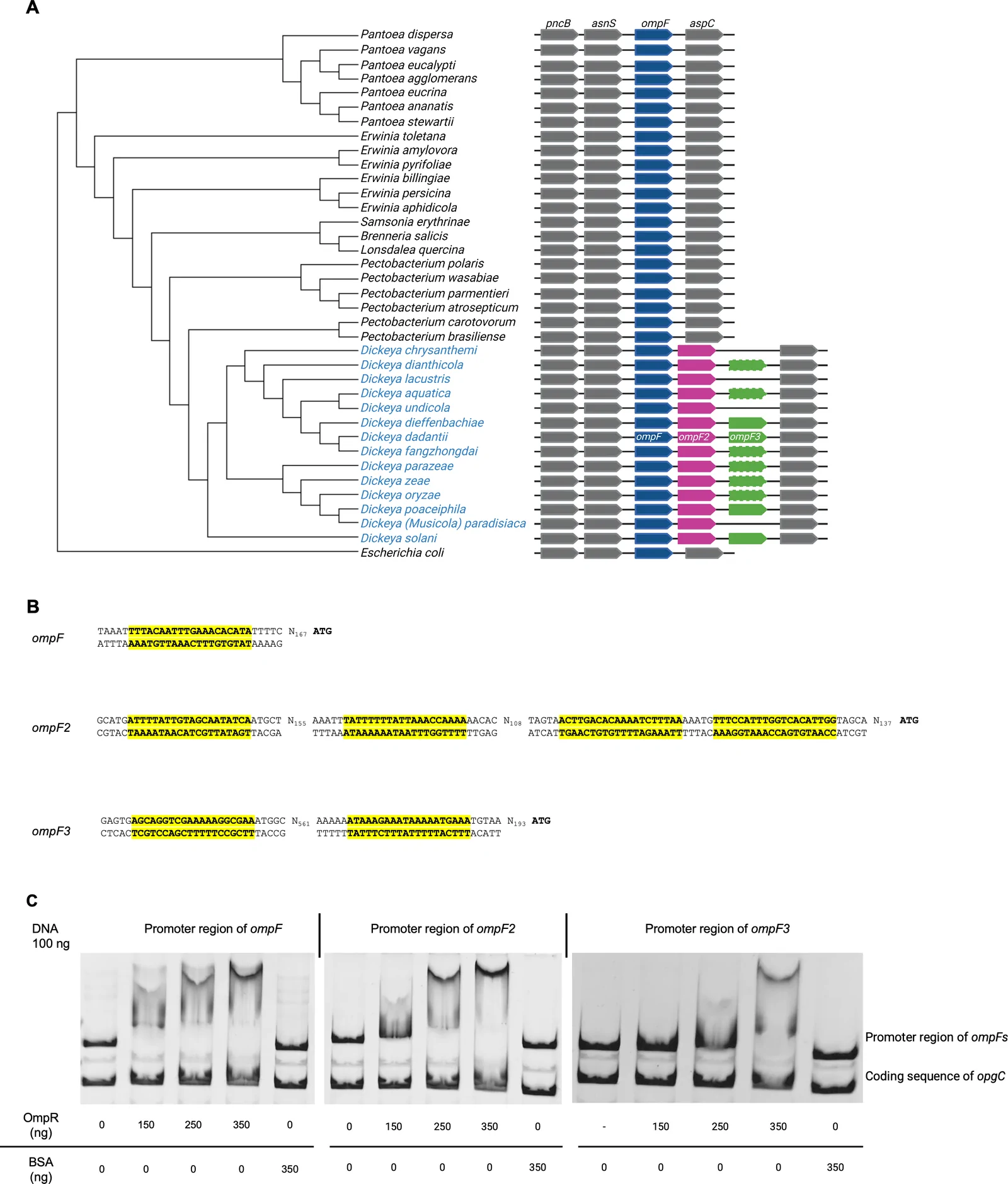
*Dickeya* sp. genomes contain three successive proteins. (**A**) Organization of the *pncB-asnS-ompFs-aspC* locus in *Escherichia coli* and in phytopathogenic bacteria from *Pectobacteriaceae* and *Erwiniaceae* family. In blue, the *ompF* gene, in pink and green, the *ompF* duplication. Rooted phylogenetic tree based on the maximum likelihood. The tree was constructed with the 16S nucleic acid sequences. (**B**) Schematic representation of the *ompFs* promoter region and *in silico* location of the OmpR binding sites (**C**) EMSA to test the binding of purified OmpR to the *ompF, ompF2,* and *ompF3* promoter regions. As a control, DNA fragments encompassing a part of the CDS sequence of *opgC* were additionally present.

Since the porin analysis showed that another band was expressed at acidic pH and no longer present in the *ompR* or *envZ* mutants, we analyzed this band, extracted from the wild-type strain grown at pH 5 (Fig. 3B), by mass spectrometry (Fig. S2). The mass spectrometry analysis revealed an outer membrane protein F encoded by ABF-0020079. Surprisingly, the genome harbored three consecutive sequences encoding outer membrane proteins: OmpF (ABF-0020081, 368 AA), OmpF2 (ABF-0020079, 369 AA), and OmpF3 (ABF0020074, 361 AA) (Fig. 4A). OmpF and OmpF2 share 70.5% of sequence identity using the ClustalW alignment program (Fig. S3). We confirmed the presence of 1, 4, and 2 putative sites of OmpR binding upstream of the *ompF*, *ompF2,* and *ompF3* genes, respectively (Fig. 4B). In addition, DNA sequences located upstream of the three porin genes interact with a purified OmpR protein as observed by electrophoretic mobility shift assay (EMSA) (Fig. 4C).

We extended our study by phylogenetic analysis of the OmpFs proteins in the related genus of the Erwiniaceae and Pectobacteriaceae families containing phytopathogens (*Brenneria, Dickeya, Pantoea, Erwinia, Pectobacterium*) as well as in other genus for comparison (*Escherichia*, *Shigella*, *Salmonella*, *Yersinia*, *Serratia*) (Table S1; Fig. 4A). The duplication of *ompF* within the *pncB-aspC* locus was observed only in members of the genus *Dickeya* (Fig. 4A) and appeared to have occurred during the emergence of the genus *Dickeya* in the common ancestor. Recently, Hugouvieux-Cotte-Pattat et al. proposed to rename *Dickeya paradisiaca* as *Musicola paradisiaca* (21); in other words, the *ompF* duplication could be linked to the emergence of the genera *Dickeya* and *Musicola*. A third *ompF* is present at this locus in many strains. Yet, it is interesting to note that *ompF3* is absent from some strains and in others, the gene is annotated as a pseudogene.

### Expression of the *ompF2* porin is pH dependent and OmpR dependent

**Fig 5:**
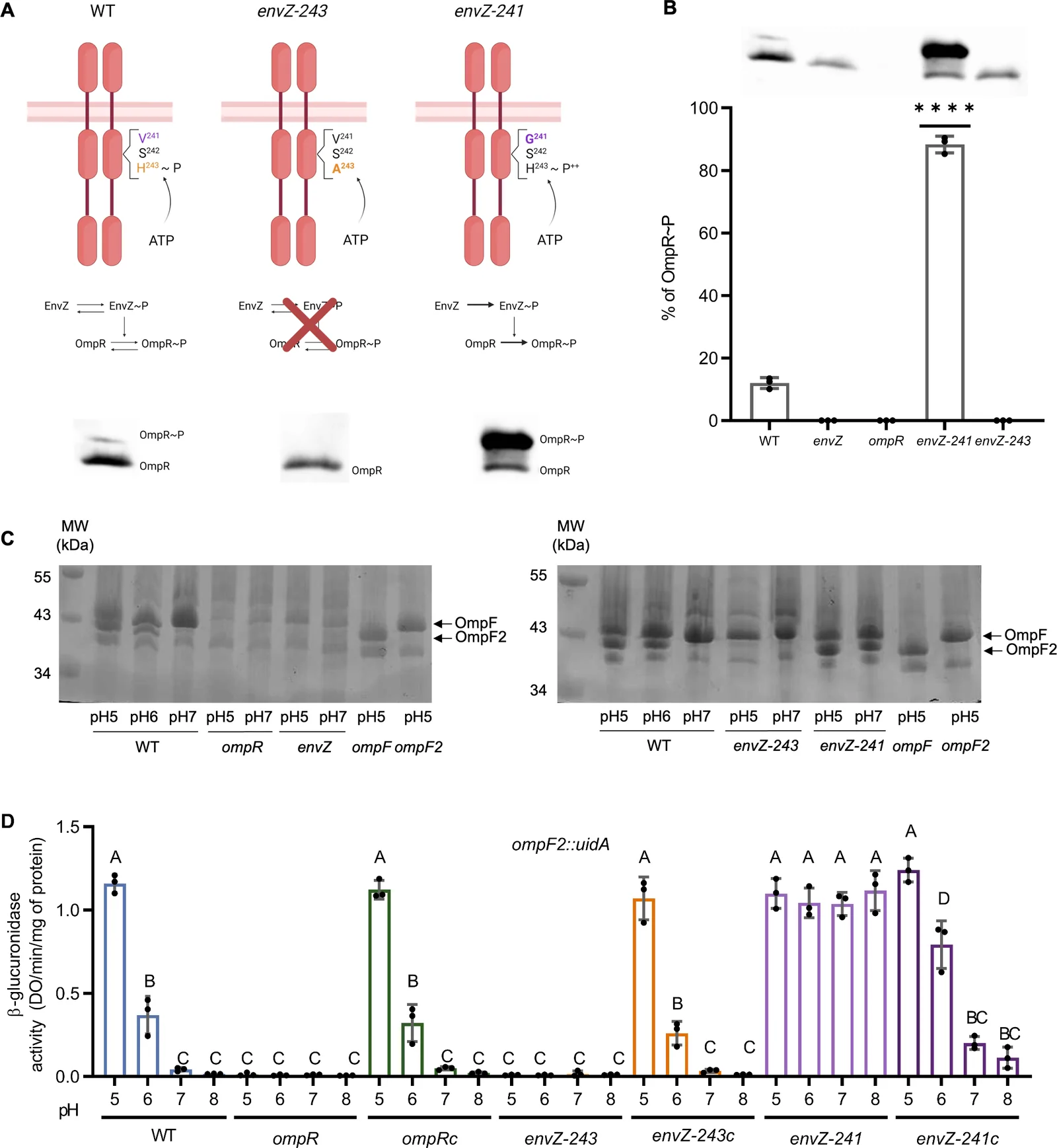
Expression of *ompF2* is pH dependent and EnvZ-OmpR dependent and repressed *in planta*. (**A**) Schematic of the different *envZ* mutants and consequence on the amount of OmpR~*P.* (**B**) Representative immunoblot of the separation of OmpR and OmpR~*P* by SDS-PAGE Phos-Tag gel after extraction bacteria from exponential growth phase culture and quantification of OmpR and OmpR~P. Both forms of OmpR were revealed by Western blot (*n* = 3 biological replicates). (**C**) Representative SDS-PAGE analysis of the outer membrane proteins showing the expression of the porins OmpFs. A gel system supplied with 4 M urea was used and stained by Coomassie staining. (**D**) Expression of *ompF2::uidA* gene fusions at different pH in different genetic backgrounds. Bacteria were grown to the mid-log phase and lysed by sonication. B-glucuronidase activity was measured with PNPU as a substrate. Specific activity was expressed as the change in OD_405_ per minute and per milligram of protein. The results are the average of three independent experiments. Groups with the same letter are not detectably different (*P* < 0.001, one-way ANOVA).

To confirm that *ompF2* is part of the OmpR regulon, we analyzed the OmpF2 amount at various pH in the wild-type strain, a null mutant strain *ompR*, as well as in the nonphosphorylatable EnvZ H243A (*envZ-243*), leading to nonphosphorylated OmpR protein and the kinase constitutive EnvZ V241G (*envZ-241*) strain (previously obtained in *Escherichia coli* (22) leading to constitutively phosphorylated OmpR protein Fig. 5A and B). Unsurprisingly, the OmpF2 amount in the outer membrane increased as the pH decreased (Fig. 5C). A previous transcriptomic analysis had shown that the corresponding gene was regulated by acidic pH (17). No OmpF2 protein was detected in the single *ompR* mutant or the inactive point mutant, while in the constitutively active mutant, the OmpF2 amount is constitutive (Fig. 5C). We confirmed this regulation at the transcriptional level, an *ompF2::uidA* bioreporter was constructed, introduced in the same genetic background, and assessed at various pH (Fig. 5D). The *ompF2* expression increased as the pH decreased in the wild-type background, while regulation is lost in strains where the EnvZ-OmpR system has been modified. Interestingly, when the transcriptional regulator OmpR is constitutively phosphorylated (*envZ-241*), the expression level and amount of *ompF2* are similar to that found in the wild-type strain at the most acidic pH (Fig. 5D). As expected, *ompF* expression was constant regardless of the genetic background or the pH (Fig. S4). These results further support our conclusions that the EnvZ-OmpR system regulates *ompF2* expression in a pH-dependent manner.

### Expression of *ompF2* is repressed in planta for full virulence

**Fig 6:**
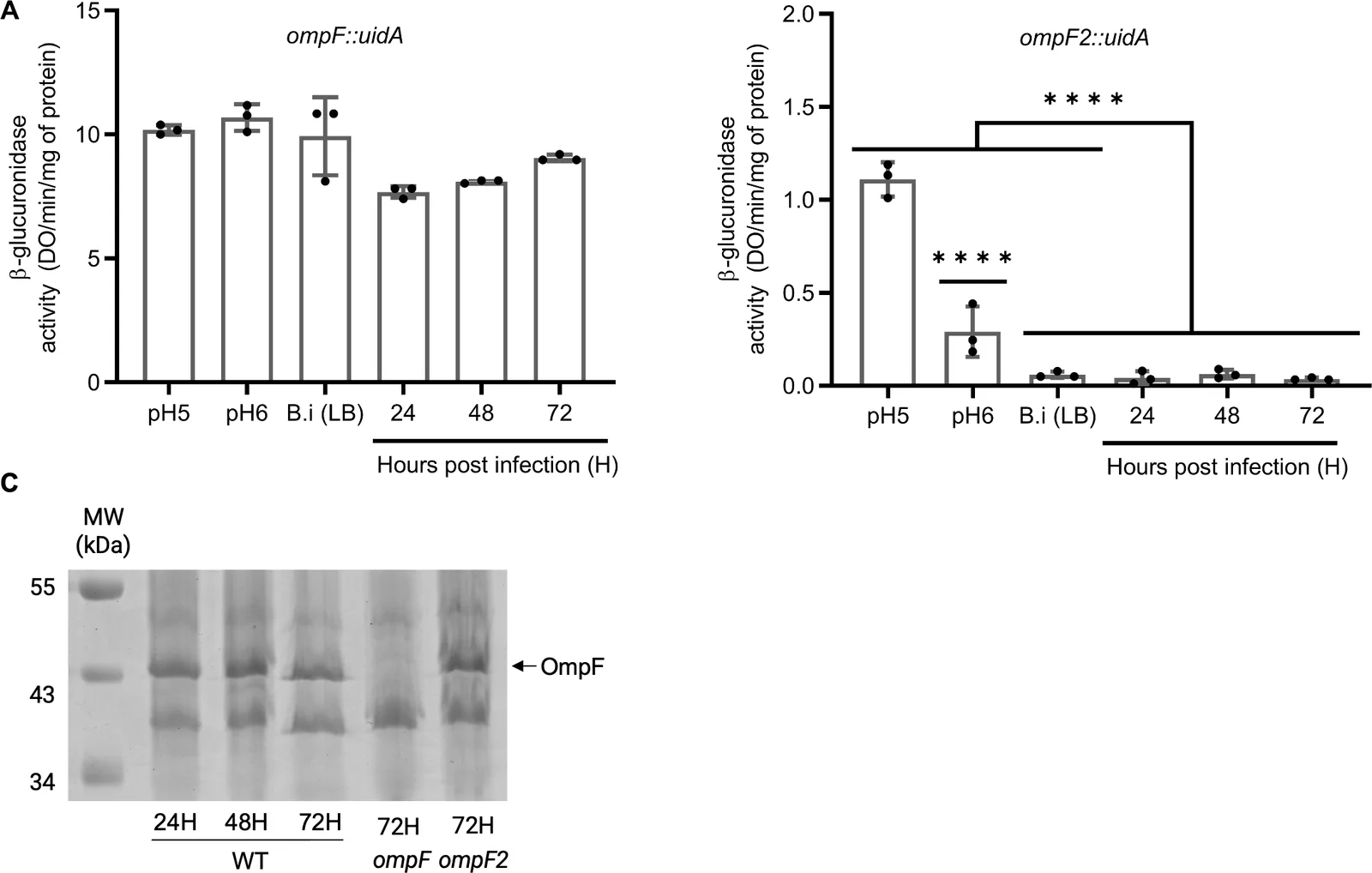
Expression of *ompF2* is repressed *in planta*. Expression of (**A**) *ompF::uidA* and (**B**) *ompF2::uidA* gene fusions in M63 medium and during the infection. Bacteria were lysed by sonication. The β-glucuronidase activity was measured with PNPU as a substrate. Specific activity was expressed as the change in OD_405_ per minute and per milligram of protein. (**C**) Representative SDS-PAGE analysis of the outer membrane proteins showing the expression of the porin OmpFs. A gel system supplied with 4 M urea was used and stained by Coomassie staining.

*In vitro*, acid pH triggered the EnvZ-OmpR system (Fig. 2J), which, in turn, upregulated *ompF2* expression (Fig. 5). *In planta*, at similar acidic pH used *in vitro*, the EnvZ-OmpR is repressed (Fig. 1D). As a consequence, both in transcriptional and protein analyses, we showed that *ompF2* is constitutively repressed while *ompF* is constitutively expressed throughout the plant’s colonization (Fig. 6A through C). As observed *in vitro*, the regulation of these two genes is solely dependent on the EnvZ-OmpR system (Fig. 3A; Fig. 5D). We hypothesized that (i) the expression of *ompF2* may result in a strong decrease in virulence and (ii) a plant component counteracts the pH stimuli preventing the activation of the EnvZ-OmpR system.

**Fig 7:**
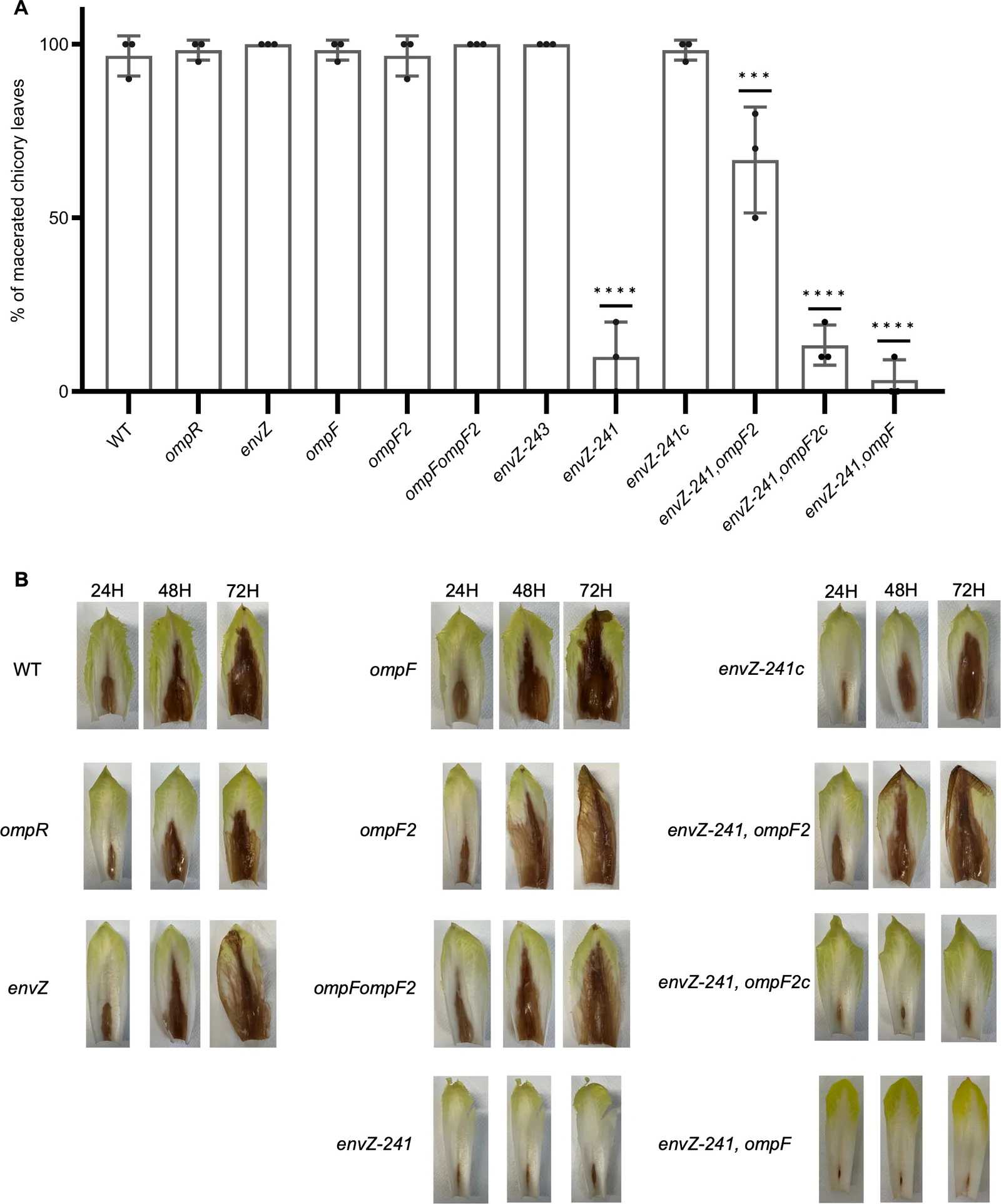
Expression of *ompF2* is deleterious for *D. dadantii* infection. (**A**) Percentage of successfully infected leaves for each strain after 72 h of infection and (**B**) representative time course of symptom development for the wild-type, *ompR*, *envZ*, *ompF*, *ompF2*, *ompF ompF2*, *envZ-241*, *envZ-241c*, *envZ-241 ompF2, envZ-241 ompF2c,* and *envZ-241 ompF* strains inoculated into chicory leaves. Virulence was monitored for 3 d.

To test this hypothesis, we assayed the virulence of the wild-type strain, a null mutant strain *ompR*, an inactive (*envZ-243*) and constitutive (*envZ-241*) active point mutants of the EnvZ-OmpR system as well as the single mutants *ompF* and *ompF2* and the double mutant *ompF ompF2* (Fig. 7A and B). The virulence in chicory leaves of the nonactivable EnvZ-OmpR mutant, the single *ompF* or *ompF2* mutants were similar to the wild type (Fig. 7A and B). In a genetic background where OmpR is constitutively phosphorylated, that is, when OmpF2 is produced, virulence is reduced by 80% compared to the wildtype (Fig. 7A). In this genetic background, if *ompF2* is deleted, the virulence is partially restored but not if *ompF* is deleted (Fig. 7A and B). Taken together, our results show that the EnvZ-OmpR system is repressed in the plant to prevent *ompF2* expression.

### The plant phenolic acids counteract the pH-dependent activation of EnvZ-OmpR to prevent OmpF2 production

**Fig 8:**
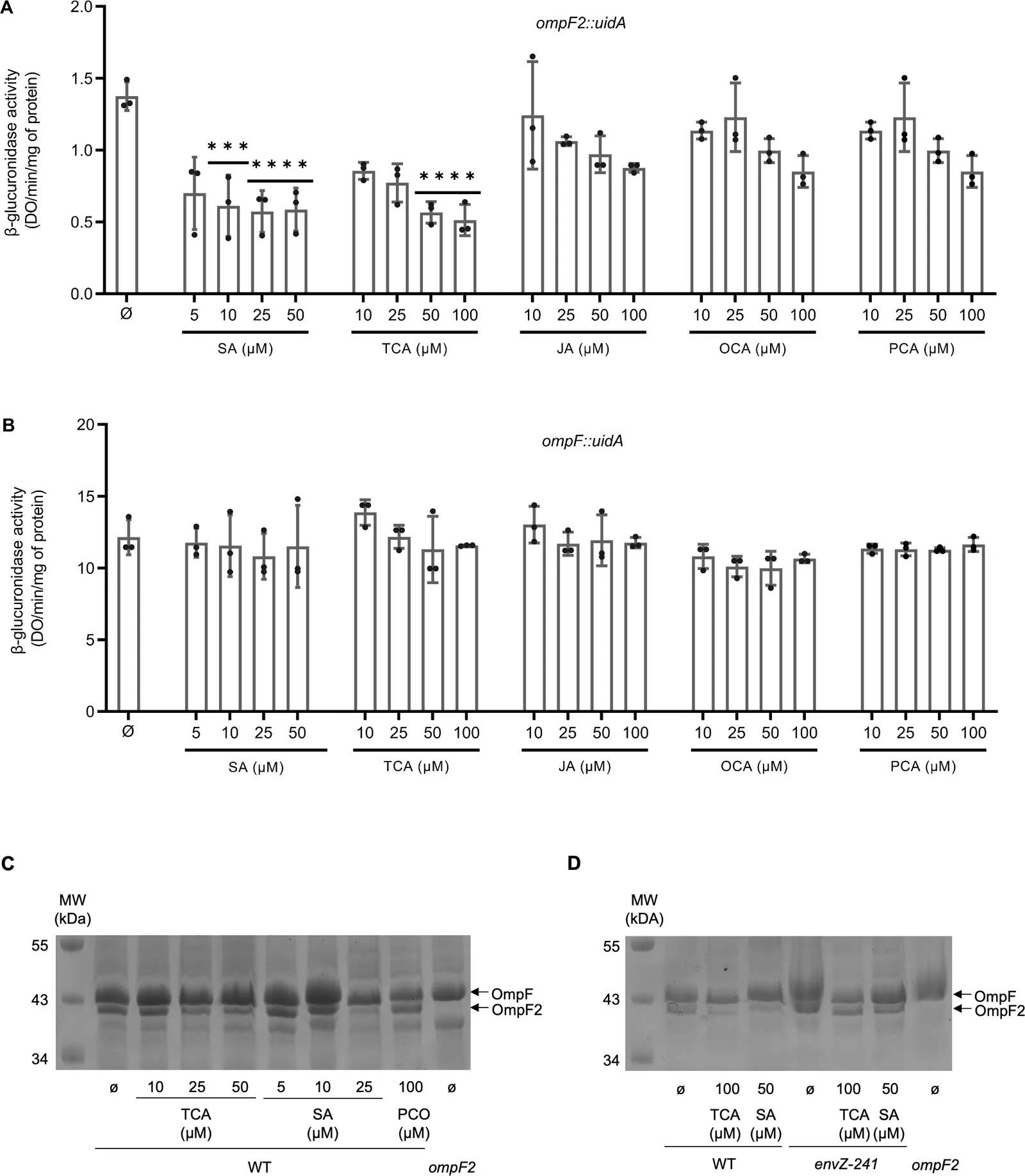
The plant phenolic acids inhibit *ompF2* expression at low pH. The plant phenolic acids inhibit the EnvZ kinase activity at low pH. Expression of *ompF2::uidA* (**A**), *ompF::uidA* (**B**) gene fusions at different concentrations of salicylic acid, cinnamic acid, jasmonic acid, o-coumaric acid, and p-coumaric acid. Bacteria were grown to the mid-log phase and lysed by sonication. The β-glucuronidase activity was measured with PNPU as a substrate. Specific activity was expressed as the change in OD_405_ per minute and per milligram of protein. The results are the average of three independent experiments.

**Fig 9:**
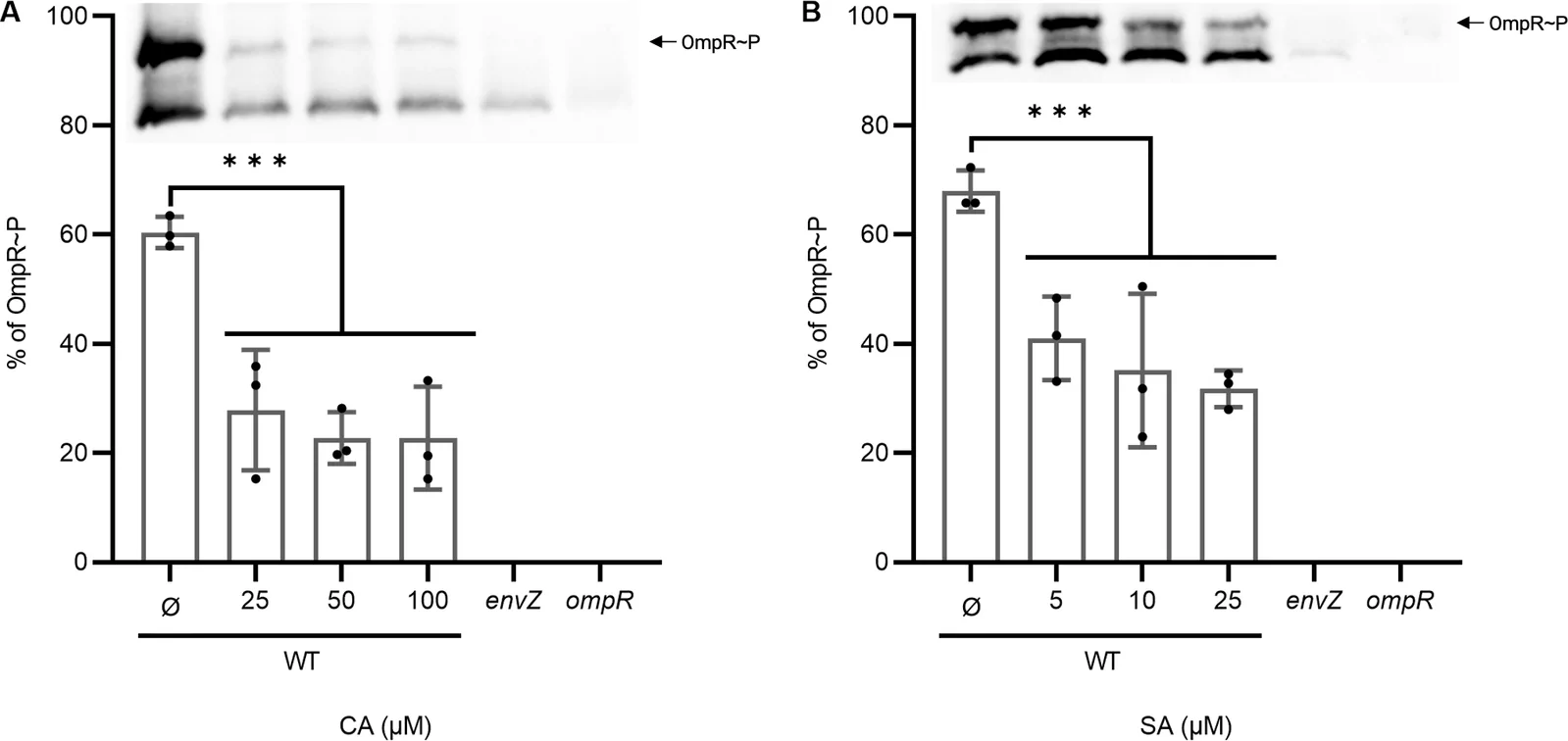
The reduction in *ompF2* expression by the phenolic acid is due to the inhibition of the EnvZ kinase activity. (**A**) and (**B**) Representative SDS-PAGE analysis of the outer membrane proteins showing the expression of the porins OmpFs. A gel system supplied with 4 M urea was used and stained by Coomassie staining. (**C**) and (**D**) Representative immunoblot of the separation of OmpR and OmpR~*P* by SDS-PAGE Phos-Tag gel after extraction of bacteria from exponential growth phase culture and quantification of OmpR and OmpR~ at different concentrations of cinnamic acid and salicylic acid. Both forms of OmpR were revealed by Western blot (*n* = 3 biological replicates).

The proposed mechanism of repression of the EnvZ-OmpR system by one or more plant-specific metabolic compounds requires that these molecules are already present when the bacterium enters the apoplast. Phenolic compounds are antioxidants that allow the regeneration of plant tissues after an injury (23 – 25). Five major compounds of this molecules family, jasmonic acid (JA), o-coumaric acid (OCA), p-coumaric acid (PCA), t-cinnamic acid (TCA), and salicylic acid (SA), are found in plants (26) and sensed by *D. dadantii* during infection (27 – 29). This prompted us to test these compounds by measuring the gene-level expression using *ompF2::uidA* bioreporter, the number of porins by extracting outer membrane proteins, and the phosphorylation level of OmpR by the Phos-Tag analysis (Fig. 8 and 9). The expression level of *ompF2* was assessed in M63 pH 5 supplemented or not with one of the five phenolic acid molecules chosen (Fig. 8A). As compared with M63 pH 5 alone, *ompF2* expression was reduced by 50% when SA was added whatever the concentration (Fig. 8A). A decrease in *ompF2* expression, which can reach 60%, is seen when adding TCA. No significant variation of *ompF2* expression was observed in the presence of JA, PCA, and OCA even at high concentrations (Fig. 8A). These observations indicate that both TCA and SA repress specifically *ompF2* since no effect of these compounds was observed with the *ompF::uidA* fusion (Fig. 8B).

To validate these results at the protein level, the same condition was used to grow the wild-type strain and analyze the porin profile (Fig. 8C). As expected, in M63 pH 5, bands corresponding to the OmpF and OmpF2 porins were observed (Fig. 8C). The OmpF2 amount decreased when different concentrations of CA or SA were added. No decrease in OmpF2 was observed when 100 µM of P-CO was added (Fig. 8C). To ensure that these compounds act via the EnvZ sensor, the porin profile of the *envZ-241* strain (constitutive phosphorylation of EnvZ) was analyzed with 100 µM of CA or 50 µM of SA. No decrease in the OmpF2 band was observed (Fig. 8D), indicating that *ompF2* repression depends on the EnvZ-OmpR TCS system. Finally, to confirm that SA and CA directly affect EnvZ-OmpR activation, we used a Phos-Tag approach to estimate the impact of these compounds on OmpR phosphorylation. Cinnamic acid and salicylic acid decreased the OmpR phosphorylated/OmpR ratio by up to 40% and 50%, respectively, and consequently the expression and synthesis of OmpF2 (Fig. 9). Taken together, these data indicate that the repression of *ompF2* and full virulence occurred by inhibition of the EnvZ-OmpR phosphorylation by CA and SA, two host compounds.

## DISCUSSION

**Fig 10:**
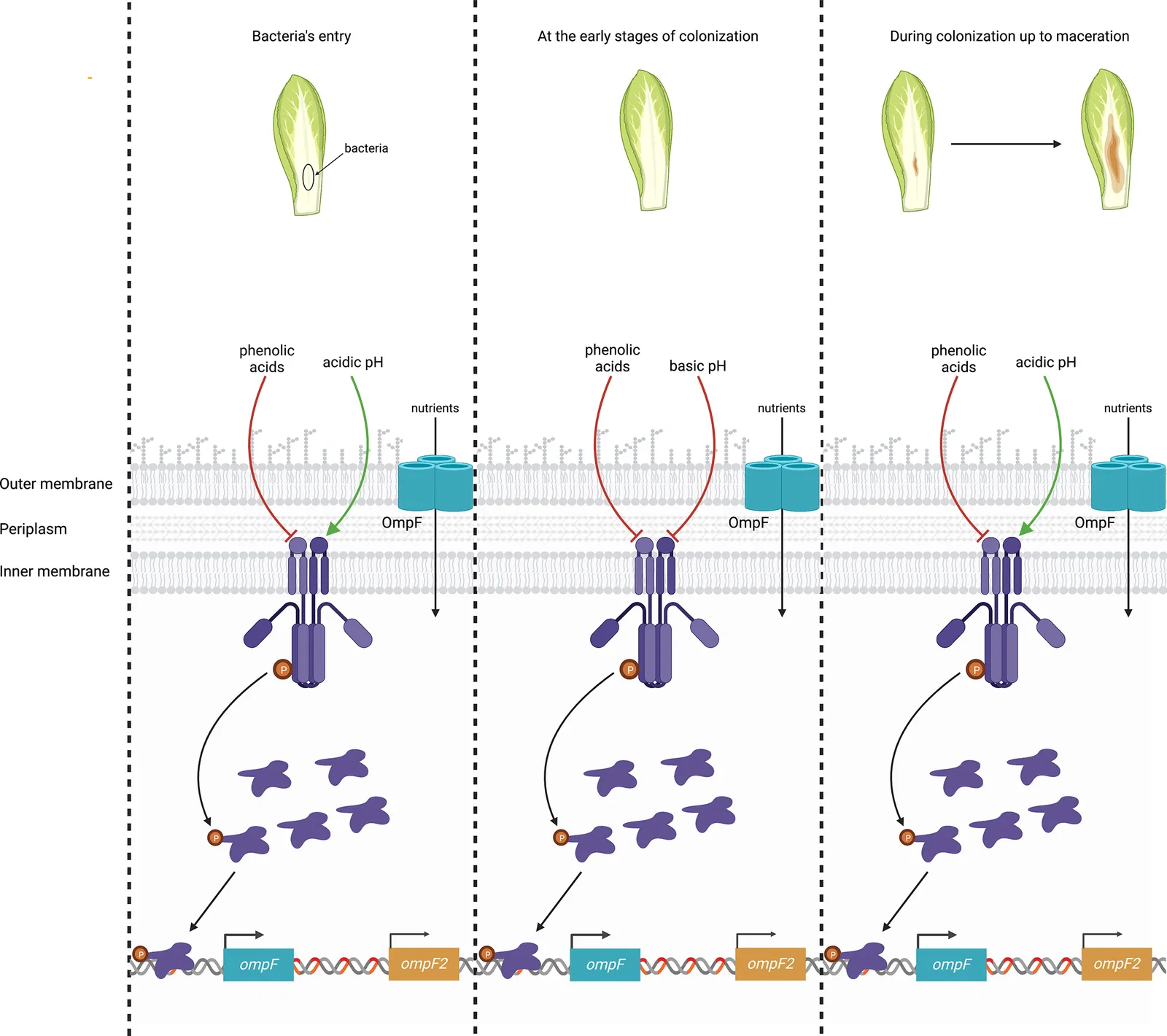
A proposed model for EnvZ-OmpR activation and *ompFs* expressions during the plant infection. (**A**) During *D. dadantii* entry in the host apoplast, the bacteria will encounter an acidic pH that should promote a high-level activation of the EnvZ-OmpR system. However, the synthesis of phenolic acids by the host cells will significantly lower the activation of the system and thus the repression of *ompF2*. (**B**) During the early phase of colonization, the bacteria will be perceived by the host cells, inducing an increase in pH and phenolic acid production. Both stimuli will maintain the low activation of EnvZ-OmpR. (**C**) From the last stage of colonization to the symptomatic phase, the apoplastic pH will be reverted to acidic but the production of phenolic acids will be maintained. Thus, the EnvZ-OmpR activation and so *ompF2* expression will be repressed throughout the infection. During this process, the *ompF* expression will be assured thanks to the low level of OmpR phosphorylation. Created with BioRender.com.

Successful colonization of the host by a pathogen relies on quick adaptation to changing environmental conditions and host defenses. TCSs are key actors in sensing and adapting to the environment. A great deal of effort is being made to understand the role of TCSs in host–pathogen interaction to address three questions: What triggers the system? Which genes are targeted? And what are the functions of these different genes? The EnvZ-OmpR system is a paradigm of the TCS (6, 30 – 35). Since its first discovery in 1977 (36), this system has been characterized as regulating the balance between two porins, OmpF and OmpC (37). The EnvZ-OmpR system is known to be triggered by osmolarity (38), pH (39), and nutrient limitation (10, 40). While the role of this system in zoopathogen virulence has been established (9 – 11, 41 – 43), in plant pathogens, this system appears to be dispensable (8, 12, 13). In *D. dadantii*, the inactivation of this system does not affect virulence *in planta* but seems to play a role in the pea aphid *Acyrthosiphon pisum* (12, 13). Our study allows us to outline a model for the role of EnvZ-OmpR during plant infection (model shown in Fig. 10). Once the bacterium enters the acidic plant apoplast through injury, the EnvZ-OmpR system (activated under acidic conditions) is silenced by the constitutive concentration of plant phenolic compound species, these concentrations being immediately enhanced by their synthesis in response to the injury (44, 45). Then, the detection of *D. dadantii*’s pathogen-associated molecular patterns (PAMPs) by the host cells leads to transient alkalization of the apoplast followed by a decrease and recovery to basal acidic pH. During all this asymptomatic step, phenolic acid synthesis is maintained in response to the presence of a pathogen (46, 47). Throughout the following symptomatic phase, in addition to PAMPs, the production of damage-associated molecular patterns (DAMPs), due to the degradation of plant cell walls, maintains the production of phenolic acids and prevents from activation of the EnvZ- OmpR system, hence the inhibition of *ompF2* expression. One can also come up with the reasonable hypothesis that the lysis of the plant cells led by the liberation of stocked phenolic compounds within plant cells (48), increasing the concentration of these molecules despite the plant cell death. Despite the absence of OmpF2, the presence of not only OmpF but also the specific porins KdgM and KdgN allows the entry of nutrients into the bacteria. KdgM and KdgN allow the passage of oligogalacturonates, which are molecules generated through the digestion of plant cell wall constituents. Interestingly, these two porins are also regulated by the EnvZ-OmpR system (20).

Our model questions the role of OmpF2 during the life cycle of *D. dadantii*. Porins are pore-forming membrane proteins that allow the diffusion of small molecules across the membrane, especially nutrients. Porin expression has been linked with sensitivity to defense molecules, antibiotics, biofilm, adhesion/invasion, and virulence (49 – 58). Knowing that *ompF2* inactivation restores virulence in the hyperphosphorylated EnvZ sensor strain (*envZ-241*), where *ompF2* is constitutively expressed (Fig. 5C), one might postulate that the expression of *ompF2* during plant colonization may allow diffusion of toxic molecules within the bacteria and may impair bacterial growth. Interestingly, OmpF has been proposed to be recognized by the host (59 – 61). Hence, one may also postulate that *ompF2* repression evades host recognition. Interestingly, duplication of the *ompF* gene belongs only to the *Dickeya* genus and is part of the emergence of this genus (Fig. 4A). In *D. dadantii* and *D. solani*, a third *ompF* was also identified (Fig. 4A). However, we were unable to detect OmpF3 in our condition (i.e., laboratory media and plants) suggesting that the gene is not expressed or at a very low level (Fig. 5C; Fig. 6C). Duplication is a process known to offer a selective advantage to the bacterium. If this advantage is not critical, then the paralog becomes a pseudogene and is eliminated (62). The absence of *ompF3* expression suggests that this gene may be undergoing clearance. The deleterious role of *ompF2* for virulence is counterintuitive. However, *Dickeya* genus bacteria are also soil bacteria. Under these conditions, the presence of both OmpF2 and OmpF in the outer membrane could be beneficial and offer a competitive advantage over other microorganisms in the field by increasing nutrient uptake, for example. Interestingly, the *in silico* structures modeled by Alpha-fold do not reveal any major structural differences. Modifications of one or two amino acids can drastically change a porin’s ability to “let through” molecules (63). Porin pore diameters vary from 6A in the more selective variants to 15A in the broader variants (64). Of particular significance, OmpF exhibits a considerable pore diameter of 11A (65). This substantial pore size plays a vital role in facilitating optimal nutrient transport, especially in situations where nutrient concentrations are low (66, 67). Further analysis is needed to determine the structural differences between OmpF and OmpF2 and their respective roles during plant infection and in the soil.

The EnvZ-OmpR system in *D. dadantii* is used in an opposite way to the other pathogens studied (10, 39, 42). In *S. enterica*, when in the host and under acidic conditions, EnvZ cannot phosphorylate OmpR while required because of acidification of the cytoplasm preventing the phosphorylation of OmpR, but a noncanonical process, involving both partners, allows OmpR dimerization to mimic the phosphorylated state and allowing interaction with an expression of the target genes as if it was phosphorylated (39). By contrast, under acidic conditions, analysis of a transcriptomic analysis (17) suggests that no cytoplasmic acidification occurs. This allows the phosphorylation of EnvZ-OmpR but the TCS remains not activated in plants with the help of phenolics; thus, the target genes were not expressed (Fig. 7A, D and E). Is this a specificity (as the noncanonical may be for *S. enterica*) of the *Dickeya* genus due to the presence of the *ompF2* porin gene or a common feature of plant pathogens? The system has been investigated only in *E. amylovora*. This pathogen of eudicots thrives in a similar environment as *D. dadantii*, that is, an acidic environment (Fig. 2A through H). However, EnvZ-OmpR is not required for virulence (8). The in silico analysis did not reveal any *ompF* duplication (Fig. 4A). So, in *E. amylovora*, is the system not required or repressed? This question remains to be elucidated.

Duplication (or even triplication) of the *ompF* gene has led to a redrawing of the regulatory network. The OmpR phosphorylated form is required for the expression of both *ompF* and *ompF2*, but only *ompF2* expression is activated by acid pH and inhibited by host phenolic acids (Fig. 8). This regulation may be explained by the presence of only one binding site in front of *ompF*, whereas four binding sites have been identified in front of *ompF2*. We can imagine that the presence of a single binding site enables on/off regulation, whereas the presence of four sites enables tight regulation of *ompF2* and avoids its expression at a time when it would be harmful to the bacterium. This model of multiple binding sites is reminiscent of the regulation of OmpF and ompC in *E. coli* (6, 68). It could be interesting to examine the affinity of the binding sites, the temporality of binding, and whether this leads to the formation of a DNA loop, as is the case for *ompF* at high osmolarity in *E. coli*.

As far as is known, it is the first TCS involved in virulence regulation by its repression. Previously, the RcsCDB system was shown to be deleterious for virulence when activated (69). The activation of the system leads to an overproduction of exopolysaccharides that make the bacterium entrapped and unable to sense its surroundings (70, 71). However, this system is neither related to virulence nor repressed during host invasion. In *D. dadantii*, the EnvZ-OmpR system is repressed by plant defense. Here, phenolic compounds synthesized and released to act against pathogens are used as a signal for the bacteria. Interestedly, salicylic acid can bind to quorum sensor receptors, interfering with the virulence of *Pectobacterium parmentieri* (72). TCSs are only considered from the point of view of activation during infection, probably because of the null mutant technique approach. Except in *D. dadantii*, the EnvZ-OmpR TCS is known to be activated during infection and regulated by different stimuli, suggesting that the system’s function has evolved to acquire new input signals (10, 15, 16, 38 – 40). Since TCSs are present in most bacteria (if not all), it would not be surprising for some of them to have shifted focus to a different set of environments and to have acquired new roles during evolution. Some of these systems can even be lost or gained during the emergence of new pathovars within the same species, allowing them to spread effectively to new hosts (73). Regarding EnvZ, the pH and osmolarity were hypothesized to have been sensed by the amino acids close to the phosphorylable histidine (74). However, in both *D. dadantii* and *E. coli,* the sequence is conserved, meaning that the reason why EnvZ in *D. dadantii* does not sense variation of osmolarity is still uncleared. Detection of cinnamic acid has been associated with the PAS (Per Arnt Sim) domain, a domain present in 33% of HKs (histidine kinases) (75, 76). However, EnvZ does not possess any PAS domain, and if specific to the *Dickeya* genus, one can hypothesize that phenolic acids are perceived by the periplasmic domain of EnvZ since it is one of the less conserved domains of sensors (77). Bacteria have co-evolved with their hosts among others to use the binding of this deleterious compound and turn it into an input signal of virulence gene regulation. This study shows an example of how TCSs can be key elements in bacterial control for everyday life, but also for the evolutionary adaptation needed to conquer new environments, particularly hosts.

## MATERIALS AND METHODS

### Bacterial strains and growth conditions

Strains, plasmids, and primers are listed in Table S2. Bacteria were cultivated in lysogeny broth (LB) at 30°C, or in minimal medium M63 supplemented with 2 g L^−1^ of glycerol as a carbon source (78). M63 acidification and alkalization were performed by adding HCL and KOH, respectively. M63 osmolarity was 330 mOsM (13). Dilution of M63 by twofold was obtained by adding H_2_O, decreasing the medium osmolarity to 170 mOsM. An increase in osmolarity to 500 and 700 mOsM was obtained by adding 0.1 and 0.2 M of NaCl. Solidification of media was obtained by adding agar at 15 g L^−1^. Antibiotics in media were used at the following concentrations: spectinomycin: 50 µg mL^−1^, chloramphenicol: 12,5 µg mL^−1^, gentamycin: 2 µg mL^−1^, and kanamycin: 25 µg mL^−1^.

### Plant infection

Potato tubers and chicory leaves were inoculated as previously described (79). Bacteria from an overnight culture in an LB medium were recovered by centrifugation and diluted in M63. For potato tubers, sterile pipette tips containing a bacterial suspension of 10^7^ cells in 5 µL were inserted into the tuber (Amandine variety). Chicory leaves, tomatoes, and green peppers were inoculated with a bacterial suspension of 10^7^ cells in 5 µL after a short incision with a scalpel. All infected plants were incubated in a dew chamber at 30°C. For pathogenicity assay, the aspect and number of infected leaves were checked after 72 h of incubation of infected chicory leaves. Area of maceration was determined with the software ImageJ (80).

### SDS-PAGE Phos-Tag gel and immunoblot analysis of OmpR phosphorylation

Phos-Tag analysis was performed on crude extracts obtained from bacteria after growth *in vivo* (M63 medium) or *in planta* after extraction from chicory leaves as described previously (81). Western blotting was performed using the rabbit anti-OmpR polyclonal antibodies at a dilution of 1: 300 and anti-rabbit secondary coupled to horseradish peroxidase at a dilution of 1: 15 000. Blots were imaged by chemiluminescent detection (SupersignalTM West Dura; Thermo Scientific, MA, USA). Phosphorylated and unphosphorylated OmpR were quantified by determining the area intensity of each band with the software ImageJ. Quantification of phosphorylated OmpR was expressed as the ratio of the phosphorylated OmpR amount divided by the sum of the phosphorylated OmpR and the unphosphorylated OmpR amounts (82).

Rabbit polyclonal antisera directed against OmpR protein was prepared by Eurogentec (Seraing, Belgium).

### Purification of His_6_-OmpR

A DNA fragment encoding the *ompR* gene *of D. dadantii* was amplified by PCR using the primers OmpR-dd-pET100-ATG-F and OmpR-dd-stop-pET100-R, cloned into a His6 tag expression vector, pET100/D-Topo (Invitrogen Life Technologies, CA, USA). The resulting His-tagged OmpR was expressed in *E. coli* BL21 (DE3), and the protein was purified by affinity chromatography according to the manufacturer’s procedure [Ni-nitrilotriacetic acid (NTA) agarose; Qiagen, Germany].

### Porin analysis

Bacteria, grown until the exponential phase, were harvested after centrifugation, washed with phosphate buffer (20 mM, pH 7.2) and lysed by sonication 4 × 45 s. After centrifugation (10,000 × *g*, 30 min), the supernatant is incubated with N-lauroylsarcosin 0.5% for 30 min at room temperature (83). Proteins were pelleted by ultracentrifugation at 100,000 × *g* for 60 min and resuspended in phosphate buffer. Loading Buffer 2× (final concentration: 250 mM Tris pH 6.5, 15% glycerol; 2.5% SDS, and 0.025% bromophenol blue) was added to 150 ng of proteins and the samples were boiled at 100°C for 5 min prior to being loaded onto a 12% acrylamide/bisacrylamide gel (final concentration 375 mM Tris pH 8.8; 0.1% SDS ; 1% APS, and 0.08% Temed). The gels were run at 25 mA with standard running buffer [0.1% (wt/vol) SDS, 25 mM Tris, and 192 mM glycine) and stained with Coomassie blue.

For mass spectrometry analysis, excised bands of Coomassie blue SDS PAGE were de-stained by five successive washes with 100 µL of a solution containing 50% of acetonitrile and 50% of 50 mM of ammonium bicarbonate. An amount of 100 µL of acetonitrile was added to the gel bands that were incubated for 20 min at room temperature under agitation at 600 rpm (Thermomixer, Eppendorf, Germany). After that, the washing effluent was discarded, and the bands were dried under flowing nitrogen gas at 40°C. For each sample, 20 µL of a 12.5 ng/µL solution of trypsin (Roche, Basel, Switzerland) was added to rehydrate the gel bands for 10 min, the supernatant was then discarded before the addition of 40 µL of 50 mM of ammonium bicarbonate. Proteolytic digestion was performed for 15 h at 37°C (Thermomixer, Eppendorf, Germany). After centrifugation at 10,000 × *g* for 5 min, the supernatant was collected and transferred to a new Protein LoBind tube (Eppendorf, Germany). An amount of 50 µL of 45% of acetonitrile and 10% of formic acid solution were added to the gel bands that were incubated for 20 min at room temperature under agitation at 600 rpm. After centrifugation at 10,000 × *g* for 5 min, the supernatant was collected and pooled with the first fraction in the Protein LoBind tube corresponding to each sample. Lastly, 50 µL of a 95% acetonitrile and 5% formic acid solution were added to the gel bands, and the supernatant was collected and also pooled with the two other fractions in the respective Protein LoBind tubes after a 20-min incubation step at room temperature under agitation at 600 rpm and a centrifugation step of 5 min at 10,000 × *g*. Finally, the pooled fractions corresponding to each sample were dried under flowing nitrogen gas at 40°C and resuspended in 25 µL of 0.1% formic acid. For each sample, 20 µL of the digest was loaded onto Evotips Pure (Evosep, Odense, Denmark) according to the manufacturer’s instructions and as previously described (84). Liquid chromatography with tandem mass spectrometry (LC-MS/MS) was performed using the Evosep One system (Evosep, Odense, Denmark) coupled to a Zeno TOF 7600 mass spectrometer equipped with an OptiFlow Turbo V ion source (SCIEX, Concord, Canada). LC was performed using the manufacturer’s 60 samples per day (60SPD) method corresponding to a gradient duration of 21 min (84). A SWATH acquisition scheme with 100 variable-size windows and 7 ms accumulation time was used. Raw data were processed with DIA-NN v1.8 (85) with a fragment ion *m/z* range of 200–1800, automatic settings of mass accuracy at the MS2 and MS1 levels and scan window, protein inference of “Genes” and a quantification strategy of “Any LC (high accuracy).” Database searches were performed using *in silico* predicted libraries from *Dickeya dadantii* UniProt canonical sequence database (UP000006859). Predicted libraries were generated using the following parameters: Trypsin/P, one missed cleavage allowed, N-term M excision, Ox(M), peptide length range: 6–30 amino acids, and precursor charge range: 2–4. Details on the library of decoy precursors generation and data matching to spectral libraries can be found in the original DIA-NN article (85).

### Electrophoretic mobility shift assay

Electromobility shift assay was performed as described previously (86). Briefly, DNA sequences upstream of *ompF*, *ompF2,* and *ompF3* and the middle of the *opgC* gene coding region (the control fragment) were amplified by PCR. A total of 100 ng of DNA fragment of interest and 100 ng of the control fragment were incubated with various amounts of OmpR-6His in gel shift buffer for 30 min at room temperature and were loaded onto a 6% acrylamide/bisacrylamide Tris/Borate/EDTA (TBE) gel. After migration, DNA was visualized by ethidium bromide staining.

### Osmolarity and pH measurements

Osmolarity and pH were measured on chicory leaves, potato tubers, green peppers, and tomatoes infected by the wild-type strain. Measurements of osmolarity and pH were carried out for 3 to 5 d post-inoculation. The pH was measured directly at the incision site using the MicroOrion pH electrode (Thermo Scientific, MA, USA). To measure osmolarity, 50 µL of macerated tissue at the incision site was retrieved and osmolarity was measured by a freezing point osmometry using the Roebling type 13 osmometer.

### Gene expression analysis

ß-Glucuronidase activity was performed on crude extracts obtained from bacteria disrupted by sonication 2 × 30 s after growth *in vivo* (M63 medium) or *in planta* after extraction from chicory leaves, as described previously (69). β-Glucuronidase activity was determined by spectrometric monitoring of the hydrolysis of PNPU (4-nitrophenyl- β-D-glucuronide) at 405 nm. Protein concentration was determined using the DS-11 Spectrophotometer (DeNovix, NC, USA).

### Comparative genomics

Genome data on phytopathogenic strains (97, including 48 *Dickeya*) were retrieved from the NCBI genome page (www.ncbi.nlm.nih.gov/genome/). When available, complete single or multiple records were preferentially used. Otherwise, original genome sequences in fasta format were downloaded and annotated. Coding sequence (CDS) prediction is followed by automatic functional assignation and manual validation for the genes of interest.

CDS detection, annotation, and comparison of porin genes were carried out using M.A.G.D.A. (Multiple Annotation of Genomes and Differential Analysis, Center for Infection and Immunity of Lille, France), a bioinformatic tool optimized to facilitate the detection of phenotype-associated nucleotide or peptidic polymorphisms by simultaneously comparing up to several hundreds of genomes. After automatic parsing of the genome files, an orthology matrix was constructed, based on the Bidirectional Best Hit results returned from tblastn queries. To prevent confusion between porin paralogues, synteny analyses were systematically run on their respective upstream and downstream flanking genes.

The phylogenic tree was built using the Phylogeny.fr website (87). The 16S nucleic acid sequences of the 37 strains were aligned using default parameters. The tree was generated using the maximum likelihood based on a bootstrapping procedure of 100 bootstraps.

### *In silico* analysis of *ompF*

The intergenic upstream DNA sequences of *ompF*, *ompF2,* and *ompF3* genes were extracted as putative promoters. Putative sites of OmpR binding of these sequences were determined by using find individual motif occurrences (88). Inputted sequences are the known binding site of OmpR upstream of *E. coli ompF* and *ompC* (89, 90) and the consensus sequence derived using Emboss cons (91).

## SUPPLEMENTAL MATERIAL

Fig. S1 spectrum.00833-23-s0001.tif The activity of EnvZ-OmpR is not regulated by osmolarity.

Fig. S2 spectrum.00833-23-s0002.tif
*D. dadantii* expresses two OmpFs.

Fig. S3 spectrum.00833-23-s0003.tif Alignment of OmpF1 and OmpF2 protein sequences from *Dickeya dadantii* strain 3937.

Fig. S4 spectrum.00833-23-s0004.tif Expression of ompF is activated but regulated by the EnvZ-OmpR system.

Table S1 spectrum.00833-23-s0005.pdf Overview of the organization of the *pncB-asnS-ompFs-aspC* locus in several bacterial species.

Table S2 spectrum.00833-23-s0006.pdf Strains, plasmids, and primers used in this study.

ASM does not own the copyrights to Supplemental Material that may be linked to, or accessed through, an article. The authors have granted ASM a non-exclusive, world-wide license to publish the Supplemental Material files. Please contact the corresponding author directly for reuse.
