# Supplementary figures and images for: Emergence of the Dickeya genus involved duplication of the OmpF porin and the adaptation of the EnvZ-OmpR signaling network
Source: Microbiol Spectr. 2023 Aug 29;11(5):e00833-23. doi: 10.1128/spectrum.00833-23 (PMC10581057; doi:10.1128/spectrum.00833-23)

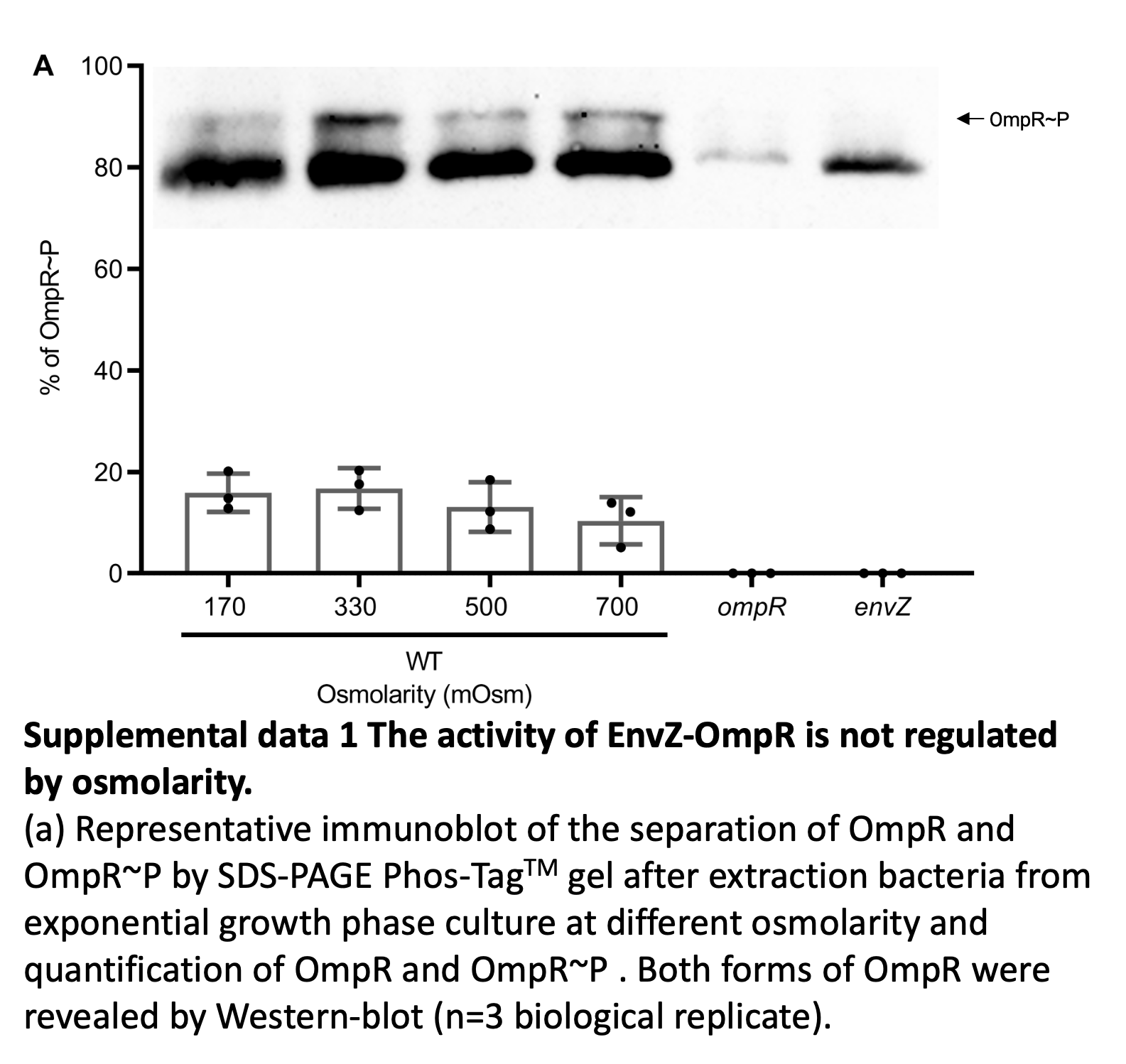

Supplement: Fig. S1 — The activity of EnvZ-OmpR is not regulated by osmolarity. [file spectrum.00833-23-s0001.tif]

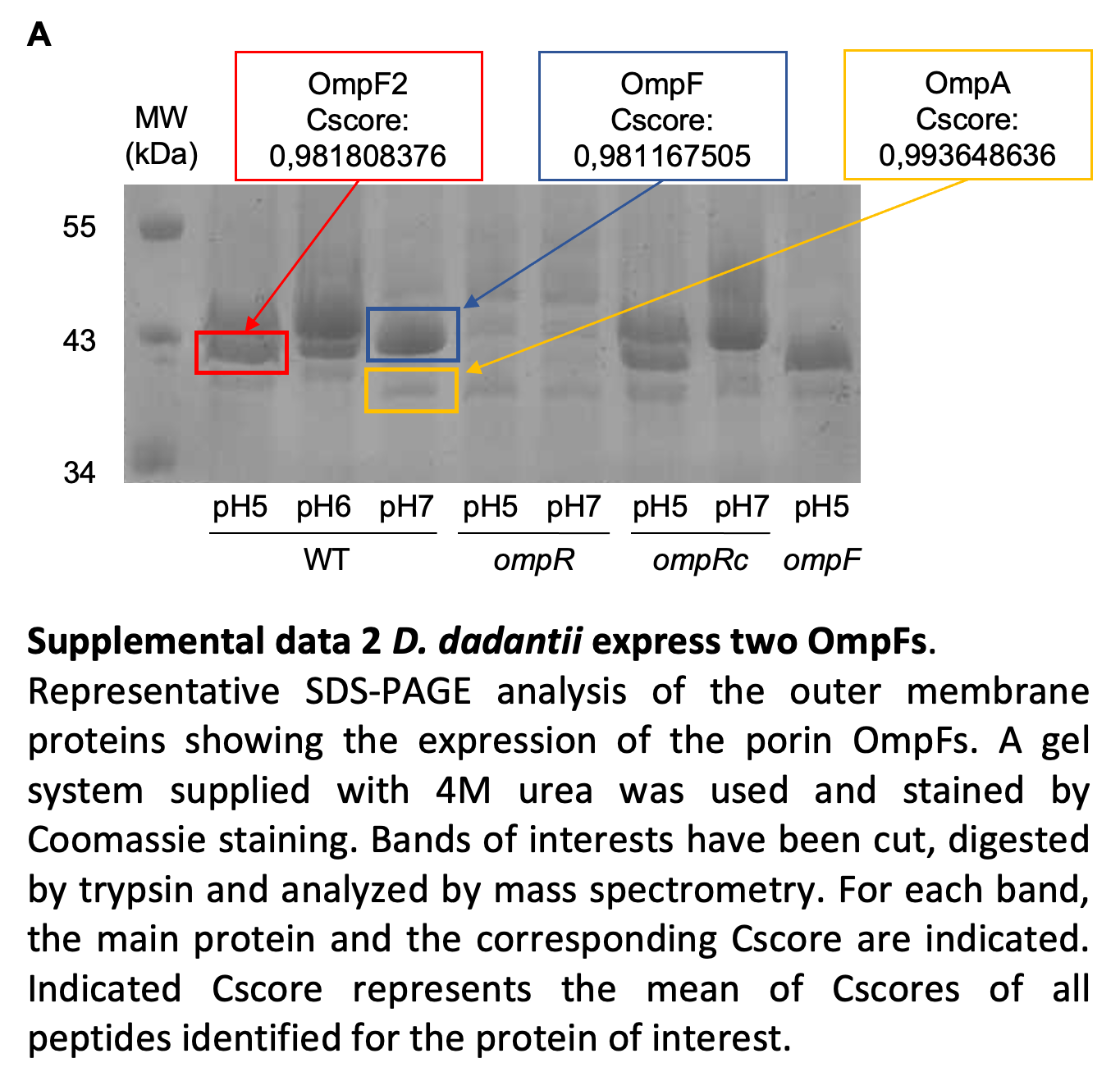

Supplement: Fig. S2 — D. dadantii expresses two OmpFs. [file spectrum.00833-23-s0002.tif]

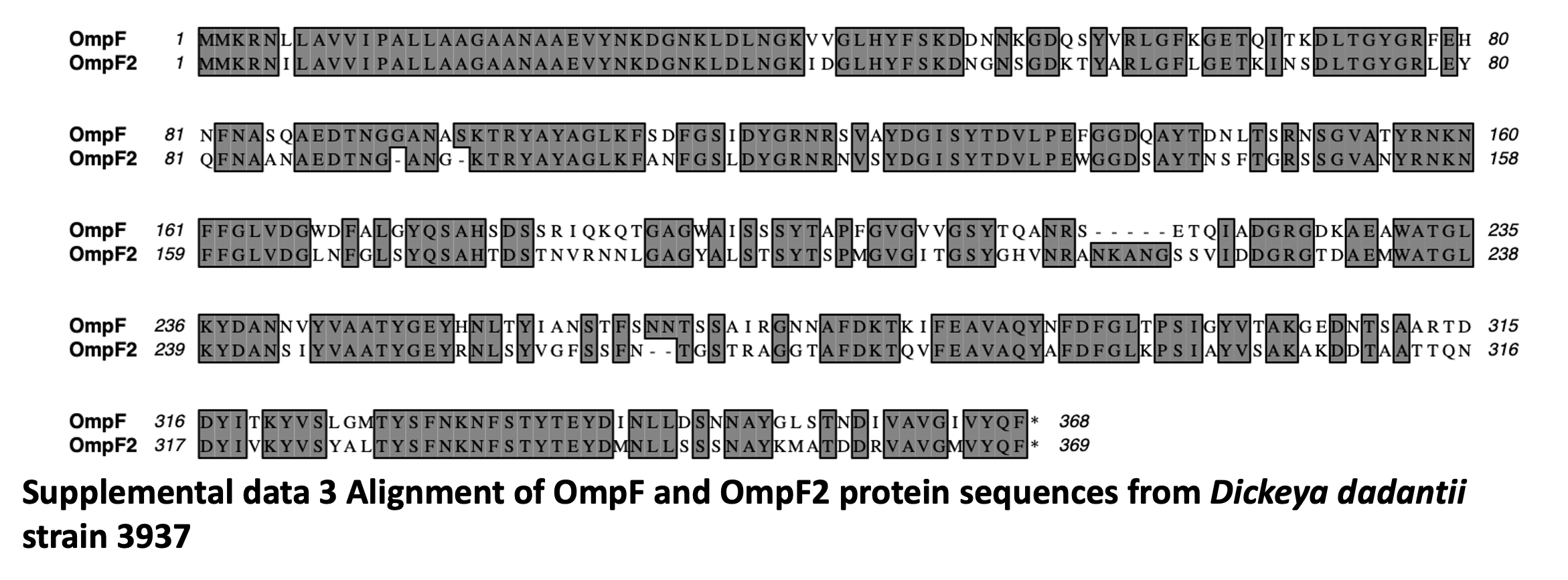

Supplement: Fig. S3 — Alignment of OmpF1 and OmpF2 protein sequences from Dickeya dadantii strain 3937. [file spectrum.00833-23-s0003.tif]

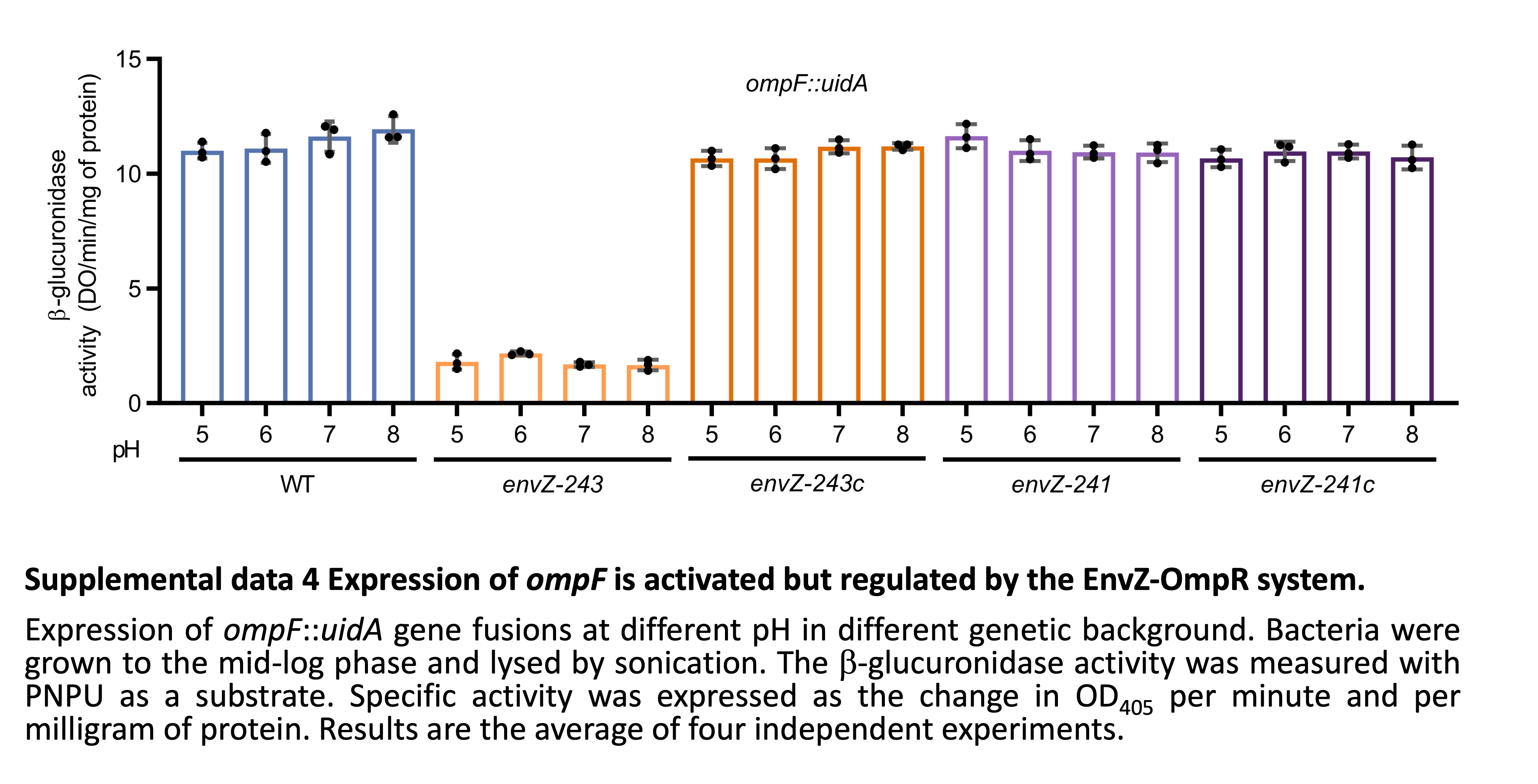

Supplement: Fig. S4 — Expression of ompF is activated but regulated by the EnvZ-OmpR system. [file spectrum.00833-23-s0004.tif]
