# Supplementary material for: Emergence of the Dickeya genus involved duplication of the OmpF porin and the adaptation of the EnvZ-OmpR signaling network
Source: Microbiol Spectr. 2023 Aug 29;11(5):e00833-23. doi: 10.1128/spectrum.00833-23 (PMC10581057; doi:10.1128/spectrum.00833-23)
Supplement: Table S1 — Overview of the organization of the pncB-asnS-ompFs-aspC locus in several bacterial species. [file spectrum.00833-23-s0005.pdf]

Supplemental Table1
Overview table of the organization of the pncB-asnS-ompFs-aspC locus in several bacterial species.

Family	Genus	species	Strain	pncB	asnS	ompF	ompF2	ompF3	aspC
Pectobacteriaceae	*Lonsdalea*	*quercina*	ATCC 29281						
	Brenneria	*salicis*	DSM 30166						
		roseae	LMG 27715						
	Dickeya	*undicola*	2B12						
			FVG1-MFV-O17						
			FVG10-MFV-A16						
		fangzhongdai	DSM 101947						
			MK7						
			Secpp 1600						
			ND14b						
			NCPPB 3274						
		dianthicola	ME23					pseudo	
			NCPPB 453					pseudo	
			IPO 980					pseudo	
			67-19						
		solani	IPO_2222						
			GBBC2040						
			RNS 05.1.2A						
		dadantii	EC3937						
			DSM 18020						
			NCPPB 3537						
			S3-1						
			NCPPB 898						
		dieffenbachiae	NCPPB_2976						
		chrysanthemi	NCPPB_516						
			NCPPB 402						
			Ech1591						
			NCPPB 3533						
		oryzae	ZYY5						
			CSL RW192						
			A5410						
		parazeae	S31						
			Ech586						
		zeae	Ech586						
			EC1						
			EC2						
			NCPPB 3531						
			A586-S18-A17						
			CE1						
			PL65						
			JZL7						
			NCPPB 2538					pseudo	
			NCPPB 3532						
			MK19						
			MS2						
			MS1						
		poaceiphila	NCPPB 569						
		lacustris	LMG30899						
		aquatica	174/2						
			DW 0440						
	Dickeya (Musicola)	*paradisiaca*	NCPPB_2511						
	Pectobacterium	*brasiliense*	SX309						
		carotovorum	PC1						
		polaris	NIBIO1006						
		parmentieri	WPP163						
		atrosepticum	JG10-08						
		wasabiae	FDAARGOS_926						
	Acerihabitans	*arboris*	SAP-6						
	Samsonia	*erythrinae*	DSM 16730						
Erwiniaceae	*Pantoea*	*stewartii*	DC283						
		ananatis	LMG20103						
		vagans	LMG 24199						
		agglomerans	C410P1						
		eucalypti	LMG 24197						
		dispersa	EGD-AAK13						
			625						
		eucrina	XL123						
		rwandensis	ND04						
	Erwinia	*amylovora*	CFBP1430						
			ATCC 49946						
		pyrifoliae	Ep1/96						
			DSM 12163						
		persicina	B64						
			NBRC 102418						
		aphidicola	ATCC 27991						
		billingiae	Eb661						
		psidii	IBSBF_435						
		tracheiphila	PSU-1						
		toletana	DAPP-PG 735						
Enterobacteriaceae	*Escherichia*	*coli*	MG1655						
			O157:H7 F8704-2						
	Shigella	*flexneri*	2a str. 2457T						
		dysenteriae	HNCMB 20080						
	Salmonella	*enterica*	14028S						
			LT2						
			SL1344						
Yersiniaceae	*Yersinia*	*enterocolitica*	IP26014						
		aleksiciae	404/81						
		pestis	KIM10						
			CO92						
		intermedia	NCTC11469						
		entomophaga	MH96						
		pseudotuberculosis	IP 31758						
		rochesterensis	ATCC BAA-2637						
		ruckeri	QMA0440						
		similis	228						
	Serratia	*rubidaea*	FDAARGOS_926						
