# Supplementary material for: Emergence of the Dickeya genus involved duplication of the OmpF porin and the adaptation of the EnvZ-OmpR signaling network
Source: Microbiol Spectr. 2023 Aug 29;11(5):e00833-23. doi: 10.1128/spectrum.00833-23 (PMC10581057; doi:10.1128/spectrum.00833-23)
Supplement: Table S2 — Strains, plasmids, and primers used in this study. [file spectrum.00833-23-s0006.pdf]

Supplementary Table 2 Strains, plasmids and primers used in this study
Amp : ampicillin resistance, Cml : chloramphenicol resistance, Gm : gentamicin resistance, Kan : kanamycin resistance,
Sp : Spectinomycin resistance.

Strains

Name	Genotype	Source or reference
	Dickeya dadantii	
EC3937	Wild-type	Laboratory collection
NFB7422	ompr::Gm	Bontemps-Gallo *et al* ., 2016
NFB7521	envZ::Gm	Bontemps-Gallo *et al* ., 2016
NFB7861	miniTn5Kan ompF-uidA	This study
NFB7876	miniTn5Sp-ompR-envZ, ompR::Gm	This study
NFB7894	ompF2::Gm	This study
NFB7895	ompF1::Kan	This study
NFB7897	ompF1F2::Gm	This study
NFB7916	miniTn5Sp-ompR-envZV241G, ompR::Gm	This study
NFB7926	miniTn5Sp-ompR-envZH243A, envZ::Gm	This study
NFB7934	miniTn5Sp-ompR-envZV241G	This study
NFB7984	miniTn5Kan-ompF-uidA, envZ::Gm	This study
NFB7985	miniTn5Kan-ompF-uidA, ompR::Gm	This study
NFB7991	miniTn5Kan-ompF2-uidA	This study
NFB7994	miniTn5Sp-ompR-envZV241G, ompR::Cml, ompF2::Gm	This study
NFB7998	miniTn5Sp-ompR-envZV241G, miniTn5Kan-ompF2, ompR::Cml, ompF2::Gm	This study
NFB9000	miniTn5Kan-ompF2-uidA, miniTn5Sp-ompR-envZV241G, envZ::Gm	This study
NFB9001	miniTn5Kan-ompF2-uidA, miniTn5Sp-ompR-envZV241G, ompR::Gm	This study
NFB9002	miniTn5Kan-ompF2-uidA, miniTn5Sp-ompR-envZH243A, envZ::Gm	This study
NFB9005	miniTn5Kan-ompF2-uidA, ompR::Gm	This study
NFB9006	miniTn5Kan-ompF-uidA, envZ::Gm, miniTn5Sp-ompR-envZ	This study
NFB9013	miniTn5Sp-ompR-envZ, miniTn5Kan-ompF2-uidA, ompR::Gm	This study
NFB9014	miniTn5Kan ompF-uidA, ompR::Gm, miniTn5Sp-ompR-envZ	This study
NFB9015	miniTn5Kan-ompF2-uidA, miniTn5Sp-ompR-envZH243A	This study
NFB9016	miniTn5Kan-ompF2-uidA , miniTn5Sp-ompR-envZV241G	This study
NFB9020	miniTn5Sp-ompR-envZ, envZ::Gm	This study
	Escherichia coli	
BL21	*ompT, hsdSB, gal, dcm*	Invitrogen
Top10F'	F-, mcrA, Δ(mrr-hsdRMS-mcrB), Φ80lacZΔM15, ΔlacX74, deoR, recA1, araD139, Δ(ara-leu)7697, galU, g	Invitrogen
S17.1 λPir	F-, RP4-2(Tc::Mu-1 ; Km::Tn7) recA1, endA1, thiE1, pro-82, hsdR17, Sm, λpir	Lorenzo *et* Timmis, 1994

References

1 Bontemps-Gallo S, Madec E, Robbe-Masselot C, Souche E, Dondeyne J, Lacroix JM. The opgC gene is required for OPGs succinylation and is osmoregulated through RcsCDB and EnvZ/OmpR in the phytopathogen Dickeya dadantii. Sci Rep. 2016;6:19619.

2 De Lorenzo V. & Timmis K. N. Analysis and construction of stable phenotypes in gram-negative bacteria with Tn5- and Tn10-derived minitransposons. Methods Enzymol. 1994;235, 386–405.

Plasmids

Name	Genotype	Source or reference
pET100/D-Topo	AmpR	Life Technologies
pUC18Not	AmpR	Yannisch-Perron *et al.,* 1985
pUC18Not-*uidA*	*uidA* , AmpR	Bontemps-Gallo *et al* ., 2014
pUTmini-Tn5-Sp	miniTn5Sp oriR6K, AmpR	*Lorenzo et al* ., 1990
pUTmini-Tn5-Kan	miniTn5Kan *oriR6K* , AmpR	*Lorenzo et al* ., 1990
pNFW457	pCR2.1 *ompR* ::Gm	Bontemps-Gallo *et al* ., 2016
pNFW480	pET100 *ompR*	Bontemps-Gallo *et al* ., 2016
pNFW515	pJET2.1 *envZ* ::Gm	Bontemps-Gallo *et al* ., 2016
209.2	pUC18Not-uidA + promotter region of *ompF*	This study
214.2	pUC18Not ompR-envZ, AmpR	This study
218.7	pUTmini-Tn5-Kan + promotter region of ompF + *uidA*	This study
240.2	pUTmini-Tn5-Sp ompR-envZ	This study
266.4	pUC18Not ompF	This study
267.8	pUC18Not ompF2	This study
271.2	pUC18Not ompF::Gm	This study
272.4	pUC18Not ompF2::Gm	This study
273.2	pUC18Not ompF-ompF2	This study
281.1	pUC18Not ompF-ompF2::Gm	This study
283.8	pUC18Not ompR-envZV241G	This study
287.16	pUT-mini-Tn5Sp ompR-envZV241G	This study
290.1	pUC18Not ompR-envZH243A	This study
298.22	pUT-mini-Tn5Sp ompR-envZH243A	This study
329.7	pUC18Not-uidA + promotter region of *ompF*2	This study
338.1	pUTmini-Tn5-Kan + promotter region of ompF2 + *uidA*	This study
339.5	pUTmini-Tn5-Kan + ompF2	This study

References

1 Yanisch-Perron, C, J Vieira, J Messing. Improved M13 phage cloning vectors and host strains: nucleotide sequences of the M13mp18 and pUC19 vectors. Gene. 1985 33(1):103-19.

2 Bontemps-Gallo S, Madec E, Lacroix JM. Inactivation of pecS restores the virulence of mutants devoid of osmoregulated periplasmic glucans in the phytopathogenic bacterium Dickeya dadantii. Microbiology. 2014;160:766–777.

3 De Lorenzo V, Herrero M, Jakubzik U, Timmis KN. Mini-Tn5 transposon derivatives for insertion mutagenesis, promoter probing, and chromosomal insertion of cloned DNA in gram-negative eubacteria. J Bacteriol. 1990;172:6568–6572.

4 Bontemps-Gallo S, Madec E, Robbe-Masselot C, Souche E, Dondeyne J, Lacroix JM. The opgC gene is required for OPGs succinylation and is osmoregulated through RcsCDB and EnvZ/OmpR in the phytopathogen Dickeya dadantii. Sci Rep. 2016;6:19619.

Name	Sequence	Source or reference
OmpF-SalI-F	GGATGTCGACCCTGGCGGAGTTCTGGAT	This study
Deb-OmpF-Sal-R	TATTGTCGACGCCGTCTTTATTGTACACTTC	This study
pUCOmpR-Hind-F	CATAAAGCTTCCACTTGCAGGCGTTTACGC	This study
pUCenvZ-Sac-R	AACTGAGCTCGATCCGCGCGACACCTATG	This study
ompF2-Xba-F	AACCTCTAGACCAATCGCTCCGAAACTCA	This study
ompF2-KpnI-R	ATATGGTACCACTTCTTTCTGGCGGCAA	This study
OmpF-SalI-F	GGATGTCGACCCTGGCGGAGTTCTGGAT	This study
OmpF-XbaI-R	AACGTCTAGAGCTACGACCAGTAAAGCTGTTG	This study
F1F2-Sal-F	GGAAGTCGACGAACCGGAAGTGGCATTTG	This study
F1F2-Xba-R	AACGTCTAGACCCATCAGCAGTCTCACATC	This study
envZV241G-F	ATGGCCGGCGGCAGCCACGAT	This study
envZV241G-R	CAGCAGCGTACGGTCATCCGC	This study
envZH243A-F	CGGCGTCAGCGCCGATCTGCGC	This study
envZH243A-R	GCCATCAGCAGCGTACGG	This study
ompF2-Xba-F2	TGTGTCTAGAGTATAGTTTATCAGTTCTAATCG	This study
ompF2-Sal-R	GTGTGTCGACGCTGTTGATTTTGGTTTCACCC	This study
OmpF-EMSA-F	CGTGAAGAACGTCTGGAACA	This study
Deb-OmpF-Sal-R	TATTGTCGACGCCGTCTTTATTGTACACTTC	This study
OmpF2-F	GTATAGTTTATCAGTTCTAATCG	This study
OmpF2-R	GAATATTACGCTTCATCATTATC	This study
ompF3-EMSA-F	CAGAGGACGCGAGGTGC	This study
ompF3-EMSA-R	CCCCCAGACGTGCATACG	This study
